# Control of skeletal morphogenesis by the Hippo-YAP/TAZ pathway

**DOI:** 10.1242/dev.187187

**Published:** 2020-11-12

**Authors:** Hannah K. Vanyai, Fabrice Prin, Oriane Guillermin, Bishara Marzook, Stefan Boeing, Alexander Howson, Rebecca E. Saunders, Thomas Snoeks, Michael Howell, Timothy J. Mohun, Barry Thompson

**Affiliations:** 1 The Francis Crick Institute, 1 Midland Rd, St Pancras, NW1 1AT London, UK; 2 EMBL Australia, Department of Cancer Biology & Therapeutics, The John Curtin School of Medical Research, The Australian National University, 131 Garran Rd, Acton, 2601, Canberra, Australia

**Keywords:** Hippo pathway, TAZ, YAP, Cartilage, Mouse embryo

## Abstract

The Hippo-YAP/TAZ pathway is an important regulator of tissue growth, but can also control cell fate or tissue morphogenesis. Here, we investigate the function of the Hippo pathway during the development of cartilage, which forms the majority of the skeleton. Previously, YAP was proposed to inhibit skeletal size by repressing chondrocyte proliferation and differentiation. We find that, *in vitro*, *Yap*/*Taz* double knockout impairs murine chondrocyte proliferation, whereas constitutively nuclear *nls-YAP5SA* accelerates proliferation, in line with the canonical role of this pathway in most tissues. However, *in vivo*, cartilage-specific knockout of *Yap*/*Taz* does not prevent chondrocyte proliferation, differentiation or skeletal growth, but rather results in various skeletal deformities including cleft palate. Cartilage-specific expression of *nls-YAP5SA* or knockout of *Lats1*/*2* do not increase cartilage growth, but instead lead to catastrophic malformations resembling chondrodysplasia or achondrogenesis. Physiological YAP target genes in cartilage include *Ctgf*, *Cyr61* and several matrix remodelling enzymes. Thus, YAP/TAZ activity controls chondrocyte proliferation *in vitro*, possibly reflecting a regenerative response, but is dispensable for chondrocyte proliferation *in vivo*, and instead functions to control cartilage morphogenesis via regulation of the extracellular matrix.

## Abstract (precis)

**Summary:** The primary role of Hippo-YAP/TAZ signalling in cartilage development is in control of tissue morphogenesis, rather than in control of cell proliferation or cell fate.

## INTRODUCTION

The Hippo signalling pathway was discovered as a potent regulator of organ size in *Drosophila*, and is conserved in mammals (Harvey and Tapon, 2007; Moya and Halder, 2019; Pan, 2007; Yu et al., 2015; Zheng and Pan, 2019). This tumour suppressor pathway consists of a core kinase cascade in which the upstream kinase MST1/2 (Hippo in *Drosophila*) phosphorylates the downstream kinase LATS1/2 (Warts in *Drosophila*), which in turn phosphorylates and inactivates the pro-proliferative transcriptional co-activators YAP (Yes-associated protein, also known as YAP1) and TAZ (transcriptional coactivator with PDZ-binding motif, also known as WWTR1), which act via TEAD-family DNA-binding transcription factors to control gene expression in response to a variety of upstream inputs (Harvey and Tapon, 2007; Moya and Halder, 2019; Pan, 2007; Yu et al., 2015; Zheng and Pan, 2019). In parallel with regulation via LATS1/2 kinases, YAP/TAZ can also be regulated by other inputs, such as direct phosphorylation by Src family kinases (Elbediwy et al., 2018; Elbediwy et al., 2016; Li et al., 2016 b; Si et al., 2017).

Genetically engineered mouse models have established that murine YAP/TAZ primarily function to promote cell proliferation and survival in many different tissues, particularly during regenerative growth or tumour formation in the intestine (Cai et al., 2015, 2010; Cotton et al., 2017; Gregorieff et al., 2015; Zhou et al., 2011), skin (Debaugnies et al., 2018; Elbediwy et al., 2016; Schlegelmilch et al., 2011; Vincent-Mistiaen et al., 2018; Zhang et al., 2011), lung (Lange et al., 2015; Lin et al., 2017), heart (Heallen et al., 2013, 2011; Leach et al., 2017; Lin et al., 2016; Monroe et al., 2019; Xin et al., 2013) and liver (Dong et al., 2007; Lee et al., 2010; Lu et al., 2018, 2010; Zender et al., 2006; Zhang et al., 2010), as well as breast ducts during pregnancy (Chen et al., 2014). In addition to controlling tissue growth, there is evidence that Hippo-YAP/TAZ signalling has a function in controlling cell fate decisions and cell differentiation during development, including specification of trophectoderm in the early blastocyst (Cockburn et al., 2013; Nishioka et al., 2009) as well as during later patterning of several tissues including the lung (Lange et al., 2015; Mahoney et al., 2014; Szymaniak et al., 2015), neural crest (Manderfield et al., 2015; Wang et al., 2016a), mesenchyme (Cotton et al., 2017), lymphatics (Cho et al., 2019) and pancreas (Rosado-Olivieri et al., 2019). Interestingly, the Hippo-YAP/TAZ pathway can have a third and distinct function in regulating morphogenesis and organ shape during development of some mammalian tissues, such as kidney (Reginensi et al., 2016, 2015, 2013) and blood vessels (Kim et al., 2017; Neto et al., 2018; Wang et al., 2017).

The role of the Hippo-YAP/TAZ pathway during development of the skeleton remains poorly understood. Endochondral skeletal development begins with formation of cartilage (chondrogenesis), the size and shape of which largely prefigures that of the resulting bony skeleton and, consequently, the size and shape of the entire body. *In vitro*, YAP was found to promote proliferation of cartilage-derived chondrocytes, suggesting a possible role of YAP in cartilage growth (Deng et al., 2016; Yang et al., 2016; Zhong et al., 2013). *In vivo*, cartilage-specific expression of wild-type YAP protein under the control of the *Col2a1* promoter in transgenic mice did not affect skeletal size or shape when heterozygous, but surprisingly reduced skeletal size when homozygous (Deng et al., 2016). Conversely, cartilage-specific conditional knockout of *Yap^flox^*^/*flox*^ with *Col2a1-Cre* was reported to increase skeletal size (Deng et al., 2016). The authors concluded that YAP primarily functions to promote early chondrocyte proliferation and inhibit chondrocyte differentiation/maturation (Deng et al., 2016). A second study reported that postnatal activation of YAP/TAZ via cartilage-specific knockout of *Mob1a*/*b* led to reduced skeletal size, owing to YAP/TAZ inhibiting both chondrocyte proliferation and differentiation/maturation (Goto et al., 2018). YAP and TAZ were proposed to inhibit differentiation/maturation via direct repression of *Sox9*, an important regulator of chondrocyte cell fate (Goto et al., 2018), a model that conflicts with the general function of YAP/TAZ as transcriptional activators.

As these initial studies did not examine complete loss- and gain-of-function of Hippo signalling during embryonic development, which requires double conditional knockouts of both *Yap* and *Taz,* or both *Lats1* and *Lats2* genes, we sought to re-examine the consequences of full activation and inactivation of the Hippo pathway in chondrocyte proliferation *in vitro* and during cartilage development *in vivo*. The resulting phenotypes are stronger than those previously reported and allow us to clarify the existing models of Hippo pathway function in cartilage. We found that YAP/TAZ are necessary and sufficient to drive chondrocyte proliferation *in vitro*, but are dispensable for chondrocyte proliferation *in vivo*. We further found that chondrocyte YAP/TAZ are not required to regulate the expression of *Sox9* and are largely dispensable for chondrocyte differentiation and subsequent endochondral ossification to produce bone. Instead, YAP/TAZ primarily function to regulate skeletal morphology, with their loss-of-function leading to abnormally shaped skeletal elements and cleft palate, and their gain-of-function generating severe skeletal malformations. These defects are driven by changes in cartilage remodelling due to dysregulation of the direct YAP/TAZ targets *Ctgf* (*Ccn2*) and *Cyr61* (*Ccn1*) as well as matrix proteases. Thus, the primary role of Hippo-YAP/TAZ signalling in cartilage development is in control of tissue morphogenesis, rather than in control of cell proliferation or cell fate.

## RESULTS

### YAP and TAZ positively regulate proliferation in primary chondrocytes *in vitro*

**Fig. 1.**
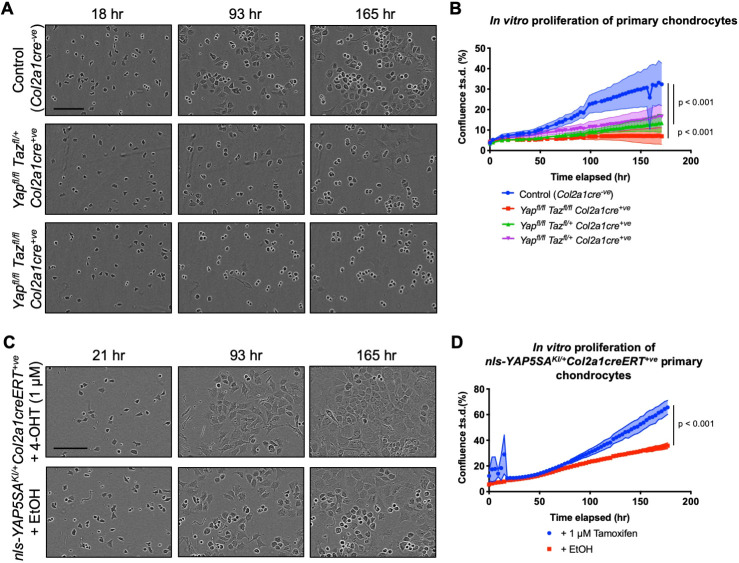
**YAP and TAZ are required for primary chondrocyte proliferation *in vitro*. (**A) Primary chondrocyte cultures from ribcages and sterna of control (*Col2a1cre^−ve^*), *Yap^fl^*^/*fl*^*Taz^fl^*^/*+*^*Col2a1cre^+ve^* or *Yap^fl^*^/*fl*^*Taz^fl^*^/*fl*^*Col2a1cre^+ve^* E17.5 pups plated at low density (3000 cells per well). (B) Proliferation, measured by confluence (percentage cell coverage) of field of view, of cultures from A. Data represent six technical replicates of primary chondrocytes derived from individual pups (biological replicates) of the indicated genotype and are representative of four independent experiments. Total biological replicates analysed were *n*=7 control (*Col2a1cre^−ve^*), *n*=4 *Yap^fl^*^/*fl*^*Taz^fl^*^/*+*^*Col2a1cre^+ve^* and *n*=4 *Yap^fl^*^/*fl*^*Taz^fl^*^/*fl*^*Col2a1cre^+ve^* E17.5 pups. Linear growth phase was measured by linear mixed model. (C) Primary chondrocyte cultures from ribcages and sterna of *nls-YAP5SA^KI^*^/*+*^*Col2a1creERT^+ve^* E17.5 pups treated with 1 μM 4-hydroxytamoxifen (4-OHT) or ethanol vehicle (EtOH) at 24 h after plating. (D) Proliferation, measured by percentage cell confluence of field of view, of cultures from C. Data represent mean of biological replicates derived from six technical replicates, treated with or without tamoxifen. *n*=3 *nls-YAP5SA^KI^*^/*+*^*Col2a1creERT^+ve^* E17.5 pups. Linear growth phase was measured by linear mixed model. Scale bars: 150 µm (A,C).

To examine the consequence of complete loss of the YAP and TAZ co-activators on *in vitro* proliferation of primary chondrocytes, we crossed *Yap^fl^*^/*fl*^*Taz^fl^*^/*+*^*Col2a1cre^+ve^* with *Yap^fl^*^/*fl*^*Taz^fl^*^/*fl*^ mice to produce litters containing *Yap^fl^*^/*fl*^*Taz^fl^*^/*fl*^*Col2a1cre^+ve^* animals. Litters were harvested at embryonic day (E)17.5 and primary chondrocytes were generated from the ribs and sterna of pups. Although control chondrocytes plated at low density (3000 cells/well of a 96-well plate) flattened and proliferated, *Yap^fl^*^/*fl*^*Taz^fl^*^/*fl*^*Col2a1cre^+ve^* chondrocytes at the same density exhibited an almost a complete arrest in proliferation and maintained a strikingly rounder morphology, consistent with the known roles of YAP/TAZ in regulating integrin adhesion in cell culture (Nardone et al., 2017) or an indication of a difference in rate of differentiation (Fig. 1A,B; Fig. S1A,B,F). Interestingly, chondrocytes with one functional *Taz* allele (*Yap^fl^*^/*fl*^*Taz^fl^*^/*+*^*Col2a1cre^+ve^*) displayed a similar pattern of proliferation arrest and morphological change, suggesting that YAP may be the primary regulator of cell proliferation and morphology in chondrocytes *in vitro*. The presence of the *Col2a1cre^+ve^* allele alone had no substantive effect on proliferation of postnatal day (P) 0 primary chondrocytes (Fig. S1A,B). Notably, apoptosis was unchanged in all genotypes (Fig. S1C-E).

We next examined the effect of increased YAP activity in primary chondrocytes, by using the cre-inducible *nls-YAP5SA* allele. This encodes a human YAP protein in which the LATS-target serines are modified to alanines, rendering the YAP protein refractory to negative regulation and cytoplasmic retention by the LATS1/2 kinases (Vincent-Mistiaen et al., 2018). A nuclear localisation signal (*nls*) further drives YAP to the nucleus, altogether resulting in the *nls-YAP5SA^KI^* allele encoding a constitutively nuclear YAP protein (Vincent-Mistiaen et al., 2018). Chondrocytes were isolated from the ribs and sterna of E17.5 pups carrying the *nls-YAP5SA^KI^* allele and the tamoxifen-inducible chondrocyte-specific *Col2a1creERT* allele. *nls-YAP5SA^KI^*^/*+*^ *Col2a1creERT^+ve^* chondrocytes treated with 1 µM of 4-hydroxytamoxifen 24 h after plating exhibited an increased rate of proliferation and more flattened morphology compared with the chondrocytes treated with vehicle control (Fig. 1C,D). Together, these data demonstrate that YAP/TAZ are necessary and sufficient to control chondrocyte proliferation and morphology *in vitro*.

### Complete loss of YAP/TAZ in chondrocytes results in lethal skeletal deformities *in vivo*

**Fig. 2.**
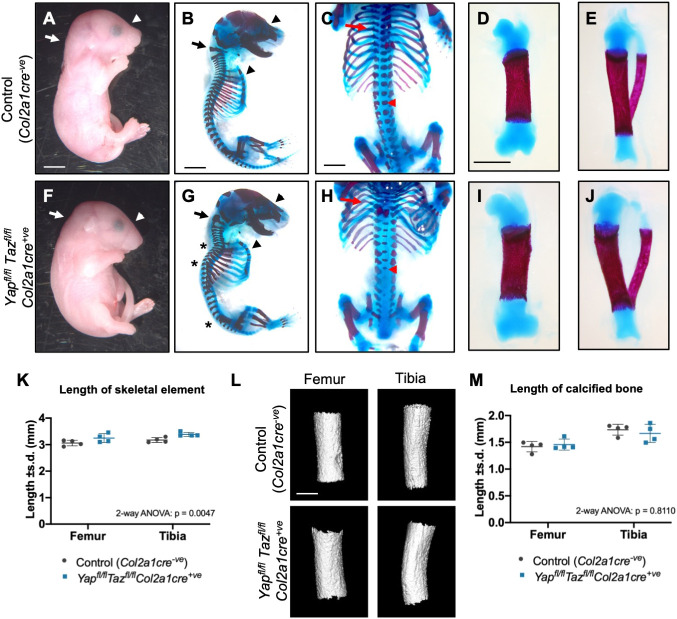
**Chondrodysplasia in *Yap*/*Taz* chondrocyte-specific knockout pups.** (A-J) Gross morphology and skeletal preparations of E17.5 control (*Col2a1cre^−ve^*) (A-E) and *Yap^fl^*^/*fl*^*Taz^fl^*^/*fl*^*Col2a1cre^+ve^* (F-J) pups. (A,F) Lateral view of E17.5 control (*Col2a1cre^−ve^*) and *Yap^fl^*^/*fl*^*Taz^fl^*^/*fl*^*Col2a1cre^+ve^* pups. White arrow indicates hunch in neck and white arrowhead indicates flattened morphology of the snout. (B,G) Lateral view of skeletal preparations of E17.5 control (*Col2a1cre^−ve^*) and *Yap^fl^*^/*fl*^*Taz^fl^*^/*fl*^*Col2a1cre^+ve^* pups with forelimbs removed. Black arrow indicates abnormal morphology of the c1 (atlas) vertebra and black arrowheads indicate the flattened rostrum of the skull and the barrel-like ribcage and curved sternum in the *Yap^fl^*^/*fl*^*Taz^fl^*^/*fl*^*Col2a1cre^+ve^* mutant compared with control. Asterisks in G indicate specific bend regions in the spine that are not present in the spine of the control. (C,H) Ventral view of skeletal preparations of E17.5 control (*Col2a1cre^−ve^*) and *Yap^fl^*^/*fl*^*Taz^fl^*^/*fl*^*Col2a1cre^+ve^* pups. Red arrows indicate the ossified (red-stained) portion of the ribs adjacent to the vertebrae emerging towards the anterior before redirecting toward the posterior in the *Yap^fl^*^/*fl*^*Taz^fl^*^/*fl*^*Col2a1cre^+ve^* mutant compared with the lateral emergence of the ossified rib in the control. Red arrowheads indicate delayed or absent ossification in the vertebrae of the *Yap^fl^*^/*fl*^*Taz^fl^*^/*fl*^*Col2a1cre^+ve^* mutant compared with control. (D,E,I,J) Isolated femur (D,I) and tibia and fibula (E,J) from skeletal preparations of E17.5 control (*Col2a1cre^−ve^*) and *Yap^fl^*^/*fl*^*Taz^fl^*^/*fl*^*Col2a1cre^+ve^* pups. Note the curvature of the tibia in J compared with E. (K) Measurements of femurs and tibias from skeletal preparations of *n*=4 control (*Col2a1cre^−ve^*) and *n*=4 *Yap^fl^*^/*fl*^*Taz^fl^*^/*fl*^*Col2a1cre^+ve^* pups. Data were analysed by two-way ANOVA, with skeletal element and genotype as the independent variables, length as dependent variable. The effect of genotype on length was significant (*P*=0.0047). (L) MicroCT volume rendered bone portions of E17.5 femurs and tibias. (M) Measurements of femurs and tibias from microCT analysis of *n*=4 control (*Col2a1cre^−ve^*) and *n*=4 *Yap^fl^*^/*fl*^*Taz^fl^*^/*fl*^*Col2a1cre^+ve^* E17.5 pups. Data were analysed by two-way ANOVA, with skeletal element and genotype as the independent variables, length as dependent variable. The effect of genotype on length was not significant (*P*=0.8110). Scale bars: 3 mm (A,B,F,); 2 mm (C,H); 1 mm (D,E,I,J); 600 µm (L).

A previous study (Deng et al., 2016) qualitatively described a modest increase in mineralised bone length and body size in *Yap^fl^*^/*fl*^*Col2a1cre^+ve^* single conditional knockout pups at late gestation. We wondered whether the presence of the YAP homologue TAZ in these animals may be sufficient to compensate for the knockout of the *Yap* gene and therefore may mask a requirement for Hippo effectors in the positive regulation of proliferation in chondrocytes *in vivo*. However, despite the extreme *in vitro* proliferation defect in primary chondrocytes isolated from E17.5 *Yap^fl^*^/*fl*^*Taz^fl^*^/*fl*^*Col2a1cre^+ve^* animals, the size of both the body and skeleton of these mutants was surprisingly normal at this stage of gestation, suggesting a profound disconnect between the *in vitro* and *in vivo* chondrocyte phenotypes for these animals (Fig. 2A-J). However, *Yap^fl^*^/*fl*^*Taz^fl^*^/*fl*^*Col2a1cre^+ve^* animals were not present at weaning, in contrast to the presence of animals carrying all other mutant allele combinations examined (including *Yap^fl^*^/*fl*^*Taz^+^*^/*+*^*Col2a1cre^+ve^*, *Yap^+^*^/*+*^*Taz^fl^*^/*fl*^*Col2a1cre^+ve^, Yap^fl^*^/*+*^*Taz^fl^*^/*fl*^*Col2a1cre^+ve^* and *Yap^fl^*^/*fl*^*Taz^fl^*^/*+*^*Col2a1cre^+ve^*), or the *Col2a1cre^+ve^* allele alone, at expected Mendelian frequencies (Fig. S2A-D). Quantification of E17.5 genotypes revealed the presence of the double homozygous mutants at Mendelian numbers (Fig. S2E), suggesting that they likely perish in the early neonatal period. On gross examination, E17.5 *Yap^fl^*^/*fl*^*Taz^fl^*^/*fl*^*Col2a1cre^+ve^* pups were hunched compared with their littermate controls, with a flattened rostrum at the dorsal surface of the snout (Fig. 2A,F). Skeletal preparations of these animals revealed subtle skeletal malformations including spine deformities, a barrel-like ribcage and sternum and a loss of convex shape of the nasal bone (Fig. 2B,C,G,H). Compared with the lateral emergence of the ribs from the spine in controls, the ribs of *Yap^fl^*^/*fl*^*Taz^fl^*^/*fl*^*Col2a1cre^+ve^* pups emerged slightly to the anterior before angling sharply down towards the posterior (Fig. 2C,H). The femur and tibia of the hindlimb of mutant pups were slightly longer than those of controls (Fig. 2D,E,I-K). The tibia furthermore exhibited a distinct bend compared with the control (Fig. 2E,J). Deng *et al.* previously described the ossified region of the limbs of *Yap^fl^*^/*fl*^*Col2a1cre^+ve^* single mutants as being longer than controls (Deng et al., 2016). We therefore scanned E17.5 animals using micro computed tomography (microCT) and measured the length of the mineralised component of the hind limb (Fig. 2L,M). There was no difference in the length of mineralisation or bone volume by this analysis, though calcium density was slightly increased in *Yap^fl^*^/*fl*^*Taz^fl^*^/*fl*^*Col2a1cre^+ve^* (Fig. 2L,M), indicating that the defects in cartilage morphogenesis do not have a large impact on skeletal size or endochondral osteogenesis in these mutants. Thus, the *Yap^fl^*^/*fl*^*Taz^fl^*^/*fl*^*Col2a1cre^+ve^* mutants did not strongly affect cartilage growth or subsequent osteogenesis, indicating that the primary requirement for YAP/TAZ is in cartilage morphogenesis. We note that phenotyping at E17.5 does not discern the direct early effects of YAP/TAZ deletion and that, to address this issue, earlier stages of development would need to be examined.

### Cleft palate in the absence of YAP/TAZ in chondrocytes

**Fig. 3.**
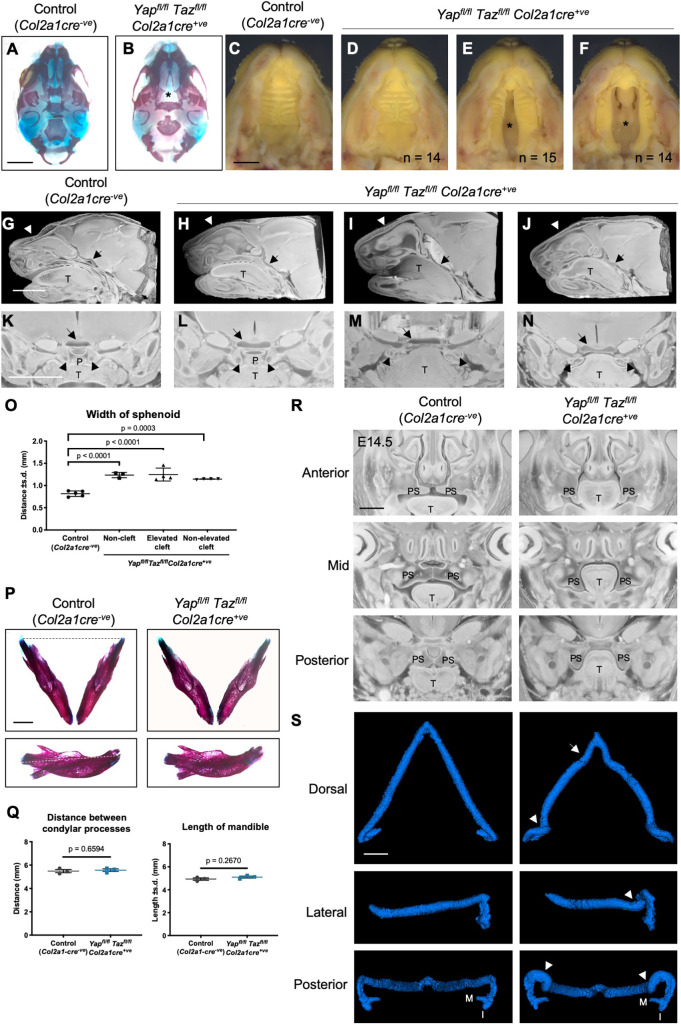
**Cleft palate in *Yap*/*Taz* chondrocyte-specific knockout pups. (**A,B) Skeletal preparations of E17.5 control (*Col2a1cre^−ve^*) and *Yap^fl^*^/*fl*^*Taz^fl^*^/*fl*^*Col2a1cre^+ve^* pups. Asterisk indicates cleft palate. (C-F) Gross morphology of the ventral surface of the palate, with lower jaw removed, showing control palate (C) and *Yap^fl^*^/*fl*^*Taz^fl^*^/*fl*^*Col2a1cre^+ve^* mutant palate (D-F), including an uncleft palate (D) or a narrow elevated (E) or wide unelevated (F) cleft palate. Asterisk indicates cleft palate, *n*=number of mutants *Yap^fl^*^/*fl*^*Taz^fl^*^/*fl*^*Col2a1cre^+ve^* E17.5 with each type of palate phenotype. (G-J) Lateral cut-away of HREM three-dimensional renderings of control (*Col2a1cre^−ve^*) (G) and *Yap^fl^*^/*fl*^*Taz^fl^*^/*fl*^*Col2a1cre^+ve^* (H-J) E17.5 pups to reveal normal morphology of the cranial base (black arrow) with the tongue (T) sitting below the intact palate (white arrowhead) in the control (G) compared with the abnormally angled cranial base (black arrow, H-J) and the tongue adjacent to the cranial base in the mutants with cleft palate (I,J). (K-N) Frontal cut-away view of the palate (P) at the level of the pterygoid process. Note the widened sphenoid cartilage (black arrow) in all mutants (L-N) and laterally rotated pterygoid processes (black arrowheads) in the cleft palate mutants (M,N). (O) Quantification of width of sphenoid. Data were analysed by one-way ANOVA followed by multiple comparisons, *P* values as indicated. HREM data derived from *n*=5 control, *n*=3 non-cleft mutant, *n*=4 elevated and *n*=4 non-elevated cleft palate mutants. (P) Isolated mandibles from skeletal preparations of E17.5 control (*Col2a1cre^−ve^*) and *Yap^fl^*^/*fl*^*Taz^fl^*^/*fl*^*Col2a1cre^+ve^* pups. Upper, dorsal view; lower, lateral view. (Q) Measurements of distance between condylar processes (dotted line in upper panel of P) and length of mandible (dotted line in lower panel of P). Data were analysed by unpaired *t*-test and were not significant (*P*=0.6594, left; *P*=0.2670, right). *n*=3 per genotype. (R) Frontal cut-away of HREM three-dimensional renderings of control (*Col2a1cre^−ve^*) and *Yap^fl^*^/*fl*^*Taz^fl^*^/*fl*^*Col2a1cre^+ve^* foetuses at E14.5 along the anterior-posterior length of the palate. The palatal shelves (PS) of 5/6 controls were elevated and partially or completed fused. All palatal shelves of five mutants remained unelevated alongside the tongue. (S) Threshold-isolated Meckel's cartilage from HREM three-dimensional renderings, representative of *n*=4 control (*Col2a1cre^−ve^*) and *n*=4 *Yap^fl^*^/*fl*^*Taz^fl^*^/*fl*^*Col2a1cre^+ve^* samples. Meckel's cartilage in the mutants (right panels) was shorter with abnormal morphology (white arrow), including an additional partial rotation at the posterior (arrowhead) before the malleus (M) and incus (I). Scale bars: 2 mm (A,B); 1.4 mm (C-F); 1.8 mm (G-N); 1 mm (P); 0.5 mm (R,S).

Interestingly, the skeletal preparations further revealed a cleft palate in some *Yap^fl^*^/*fl*^*Taz^fl^*^/*fl*^*Col2a1cre^+ve^* animals, which would contribute to their neonatal lethality (Fig. 3A,B). The palate initially develops as bilateral palatal shelves growing horizontally from the maxilla between E10.5 and E12.5. At E13.5, the palatal shelves lie vertically alongside the tongue, and undergo a rapid process of elevation at around E14.0, which requires the tongue to drop by the movement of the lower jaw opening. At E14.5, the palatal shelves are positioned vertically above the tongue and continue to grow toward the midline where they meet and fuse by around E15.5. Cleft palate can occur by failure of any of these stages of development. Examination of the palate by gross dissection at E17.5 indicated that, compared with littermate controls (Fig. 3C) *Yap^fl^*^/*fl*^*Taz^fl^*^/*fl*^*Col2a1cre^+ve^* mutants displayed either no cleft (Fig. 3D), a narrow cleft with elevated palatal shelves (Fig. 3E) or a wide cleft with unelevated palatal shelves (Fig. 3F), at approximately even incidence. To determine the developmental origin of the cleft palate phenotype, we performed high resolution episcopic microscopy (HREM) on heads of E17.5 pups and generated three-dimensional models of the head by volume rendering. Examination of lateral cutaways of the volume-rendered HREM images revealed that the shape of the endochondral bones of the cranial base was deformed in all *Yap^fl^*^/*fl*^*Taz^fl^*^/*fl*^*Col2a1cre^+ve^* mutants (Fig. 3G-J). Of the three bones that contribute to the palate, the palatal processes of the palatine and maxillary bones are both derived from intramembranous ossification rather than endochondral ossification, leading us to examine the region of the pterygoid process, which develops by secondary endochondral ossification. The basisphenoid cartilage of the cranial base was wider in all three classes of mutant palates compared with control, whilst the pterygoid processes angled towards the midline in controls and non-cleft mutants (Fig. 3K,L) but angled laterally in both classes of cleft mutants (Fig. 3M,N). The basisphenoid measured significantly wider in the frontal plane (Fig. 3O). The tongue was tightly wedged in the palatal space in the cleft palate mutants (Fig. 3I,J,M,N), suggesting that the cleft palate phenotype may be a secondary consequence of the tongue physically impeding the elevation and/or closure of the palatal shelves. This can occur owing to a failure of the lower jaw to drop during palate elevation because of changes in the morphology of the lower jaw, such as a shortening of the mandibles (Ricks et al., 2002). Though the mandibles themselves develop through intramembranous ossification, their growth is guided by the rod-like Meckel's cartilage around which they grow. However, the mandibles of *Yap^fl^*^/*fl*^*Taz^fl^*^/*fl*^*Col2a1cre^+ve^* mutants with the most severe (unelevated) cleft at E17.5 were indistinguishable from controls (Fig. 3P,Q). We therefore examined during and directly after the elevation stage of palate development using HREM at E14.5 and E15.5. At E14.5, the palatal shelves of four out of five control (*Col2a1cre^−ve^*) foetuses were fully elevated, with the remaining foetus having one palatal shelf unelevated, in contrast to all five *Yap^fl^*^/*fl*^*Taz^fl^*^/*fl*^*Col2a1cre^+ve^* mutants having unelevated palatal shelves (Fig. 3R). Meckel's cartilage was extracted using object thresholding from the E14.5 HREM images, which revealed abnormal morphology and anterior-posterior shortening of Meckel's cartilage in all five *Yap^fl^*^/*fl*^*Taz^fl^*^/*fl*^*Col2a1cre^+ve^* mutants compared with the five control (*Col2a1cre^−ve^*) Meckel's cartilages (Fig. 3S). By E15.5, the four mutants examined had elevated palatal shelves (*n*=2) or one shelf elevated (*n*=2), compared to fully elevated palatal shelves in three controls (*Col2a1cre^−ve^*; Fig. S2F). These data suggest that the cleft palate observed in two thirds of *Yap^fl^*^/*fl*^*Taz^fl^*^/*fl*^*Col2a1cre^+ve^* E17.5 pups may be caused by abnormal morphology of Meckel's cartilage, and therefore the lower jaw, at the developmental timepoint crucial for palate elevation, which may prevent the tongue from lowering and lead to a delay in or prevention of elevation. Interestingly, the defects in Meckel's cartilage can be overcome in *Yap^fl^*^/*fl*^*Taz^fl^*^/*fl*^*Col2a1cre^+ve^* animals, resulting in normal mandibular morphology at E17.5 and even normal palate morphology in some mutants. Thus, loss of YAP and TAZ causes defects in Meckel's cartilage as well as the cartilages of the cranial base, leading to defects in palate closure and resulting in cleft palate and neonatal lethality.

### YAP/TAZ are not required for cell proliferation in the cartilage growth plate *in vivo*

**Fig. 4.**
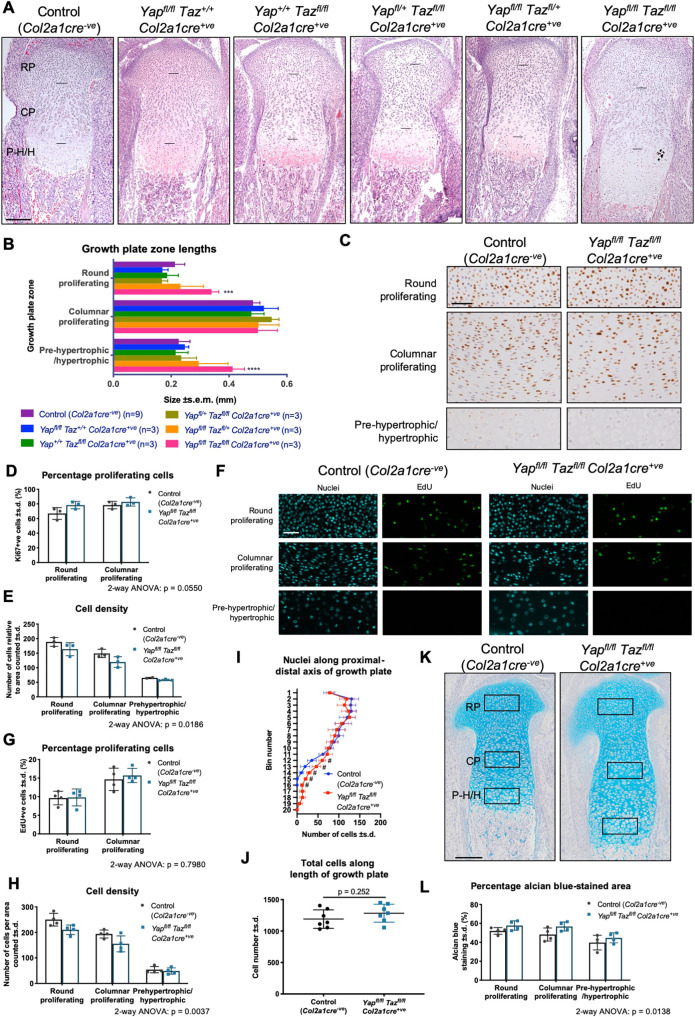
**Elongated growth plate and no change to proliferation in *Yap*/*Taz* chondrocyte-specific knockout pups. (**A) H&E-stained histological sections of the proximal growth plate of the tibia of E17.5 pups. Horizontal black lines demarcate the borders of the round proliferating (RP), columnar proliferating (CP) and pre-hypertrophic/hypertrophic (P-H/H) zones. Note the elongated growth plate in the *Yap^fl^*^/*fl*^*Taz^fl^*^/*fl*^*Col2a1cre^+ve^* mutants compared with all other genotypes. (B) Measurements of growth plate zones, analysed by two-way ANOVA followed by Dunnett's multiple comparisons test of each genotype relative to control (*Col2a1cre^−ve^*). *n* is indicated in figure legend. ***P=0.0004; ****P<0.0001. (C) Immunostaining for Ki67 in the zones of the proximal growth plate of the tibia in *n*=3 control (*Col2a1cre^−ve^*) and *n*=3 *Yap^fl^*^/*fl*^ *Taz^fl^*^/*fl*^*Col2a1cre^+ve^* E17.5 pups. (D,E) Quantification of proliferating cells (D) and cell density (E) of samples from C. Data were analysed by two-way ANOVA, with growth plate zone and genotype as the independent variables, percentage proliferating cells (D) or cell number (E) as the dependent variable. The effect of genotype on proliferation (D) was not significant (*P*=0.0550) and effect of genotype on cell density (E) was significant (*P*=0.0186). *n*=3 sections per growth plate per genotype. (F) Fluorescent immunostaining for nuclei (cyan) and EdU (green) in the zones of the proximal growth plate of the tibia in *n*=4 control (*Col2a1cre^−ve^*) and *n*=4 *Yap^fl^*^/*fl*^*Taz^fl^*^/*fl*^*Col2a1cre^+ve^* E17.5 pups collected 2 h after injection of dams with EdU. *n*=3 sections per growth plate per genotype. (G,H) Quantification of the percentage of proliferating cells (G) and cell density (H) of samples from F. Data were analysed by two-way ANOVA, with growth plate zone and genotype as the independent variables, percentage proliferating cells (G) or cell number (H) as the dependent variable. The effect of genotype on proliferation (G) was not significant (*P*=0.7980) and effect of genotype on cell density (H) was significant (*P*=0.0037). (I,J) Quantification of cell number along longitudinal length of growth plate, either binned (I) or total (J). Data were analysed by multiple *t*-tests and corrected for multiple testing (I; # indicates adjusted *P*<0.01) or unpaired *t*-test (J; *P*=0.252). *n*=7 per genotype. (K) Alcian Blue staining of proximal growth plate of the tibia in *n*=4 control (*Col2a1cre^−ve^*) and *n*=4 *Yap^fl^*^/*fl*^*Taz^fl^*^/*fl*^*Col2a1cre^+ve^* E17.5 pups. Boxes, from top to bottom, indicate regions from round proliferating, columnar proliferating and pre-hypertrophic/hypertrophic zones used for quantifying Alcian Blue staining. (L) Quantification of percentage area occupied by ECM (Alcian Blue-stained area) per boxed region from K. Data were analysed by two-way ANOVA, with growth plate zone and genotype as the independent variables, percentage area Alcian Blue-stained as the dependent variable. The effect of genotype on percentage Alcian Blue-stained area was significant (*P*=0.0138). Scale bars: 200 µm (A,K); 50 µm (C,F).

To investigate the cellular basis for the YAP/TAZ loss-of-function phenotype in cartilage, we focused on the growth plate of the proximal tibia, a commonly examined cartilage structure for the study of chondrocyte proliferation and differentiation *in vivo*. The growth plate provides a pseudotemporal snapshot of chondrogenesis (Li et al., 2016a), enabling simultaneous examination of the different stages of chondrocyte development from round proliferating chondrocytes to columnar proliferating chondrocytes, after which they stop proliferating and undergo hypertrophy to become pre-hypertrophic, then hypertrophic and finally terminally differentiated chondrocytes. Some terminally differentiated chondrocytes undergo apoptosis, while the remainder transdifferentiate into osteoblasts (Yang et al., 2014; Zhou et al., 2014) to make way for mineralised bone. Stage-specific defects in chondrogenesis can be inferred from the variation in size of growth plate zones, gene expression domains or regions of proliferation or apoptosis (Hallett et al., 2019). *Yap* and *Taz* are expressed throughout the growth plate at all stages of chondrogenesis, with both nuclear and cytoplasmic immunostaining apparent throughout (Fig. S3A,B). Haematoxylin and Eosin (H&E) staining of sections of E17.5 control (*Col2a1cre^−ve^*), single mutants (*Yap^fl^*^/*fl*^*Taz^+^*^/*+*^*Col2a1cre^+ve^*, *Yap^+^*^/*+*^*Taz^fl^*^/*fl*^*Col2a1cre^+ve^*), animals retaining one intact copy of either *Yap* or *Taz* (*Yap^fl^*^/*+*^*Taz^fl^*^/*fl*^*Col2a1cre^+ve^* and *Yap^fl^*^/*fl*^*Taz^+^*^/*fl*^*Col2a1cre^+ve^*, respectively) and the double mutant (*Yap^fl^*^/*fl*^*Taz^fl^*^/*fl*^*Col2a1cre^+ve^*) revealed that only the growth plate of the double mutant was changed in total length (Fig. 4A) and length of each growth plate zone (Fig. 4B) compared with the control. The presence of the *Col2a1cre^+ve^* allele alone also did not affect total growth plate length or the size of individual zones (Fig. S4). In contrast, there was an expansion of the round proliferating zone and the pre-hypertrophic/hypertrophic zone in the *Yap^fl^*^/*fl*^*Taz^fl^*^/*fl*^*Col2a1cre^+ve^* growth plates compared with control, suggesting defects in these stages of chondrogenesis (Fig. 4A,B). Proliferation, as measured by the percentage of Ki67-staining cells (compared with total cells marked by Eosin staining of the nuclei) in each chondrocyte zone of the growth plate, was unchanged in *Yap^fl^*^/*fl*^*Taz^fl^*^/*fl*^*Col2a1cre^+ve^* samples compared with control (Fig. 4C,D). However, the density of cells per region counted was reduced in the mutants (Fig. 4E). To confirm these results, proliferation was measured by EdU incorporation versus total DAPI-stained nuclei, and again there was no change although cell density was reduced (Fig. 4F-H). TUNEL staining revealed no apoptosis in either control or mutant samples (Fig. S3C), demonstrating that the difference in cell density in *Yap^fl^*^/*fl*^*Taz^fl^*^/*fl*^*Col2a1cre^+ve^* samples is not due to loss of cells. Binning the number of cells down the longitudinal length of the growth plate in control and *Yap^fl^*^/*fl*^*Taz^fl^*^/*fl*^*Col2a1cre^+ve^* samples revealed approximately equivalent cell numbers in the proximal two thirds of the growth plate and a slight increase in cells in the mutant in the most distal regions (Fig. 4I); however, the overall total number of cells was not significantly different (Fig. 4J), suggesting no extensive delay in differentiation. These experiments show that in the mutants there is lower cell density, but approximately equivalent numbers of cells and equivalent rates of proliferation, across a greater area, with no change in apoptosis. Together these results suggest that there is an increase in extracellular space (i.e. extracellular matrix; ECM) throughout the mutant growth plate as well as an expansion in length of the hypertrophic zone and, accordingly, staining with Alcian Blue confirmed an increase in total area of matrix and increased percentage of matrix versus cellular material in each region (Fig. 4K,L). As cartilage morphogenesis involves the stage-specific and timely production and degradation of ECM proteins, the function of YAP/TAZ in chondrogenesis may relate to synthesis and/or remodelling of the ECM, rather than its canonical role in promoting cell proliferation.

### Constitutive activation of YAP in chondrocytes does not affect cell proliferation but causes severe skeletal deformities *in vivo*

**Fig. 5.**
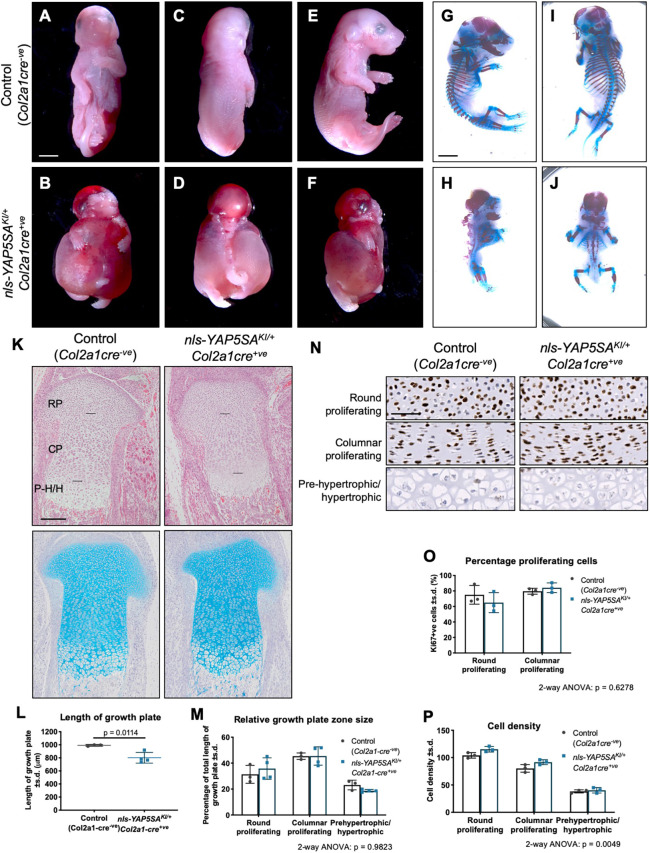
**Severe chondrodysplasia in pups with constitutively nuclear YAP in chondrocytes. (**A-F) Ventral (A,B), dorsal (C,D) and lateral (E,F) views of control (*Col2a1cre^−ve^*) (A,C,E) and *nls-YAP5SA^KI^*^/*+*^*Col2a1cre^+ve^* (B,D,F) E17.5 pups. (G-J) Lateral (G,H) and dorsal (I,J) views of skeletal preparations of control (*Col2a1cre^−ve^*) (G,I) and *nls-YAP5SA^KI^*^/*+*^*Col2a1cre^+ve^* (H,J) E17.5 pups. (K) H&E- (upper) and Alcian Blue- (lower) stained histological sections of the proximal growth plate of the tibia of *n*=3 control (*Col2a1cre^−ve^*) and *n*=3-4 *nls-YAP5SA^KI^*^/*+*^*Col2a1cre^+ve^* E17.5 pups. Horizontal black lines demarcate the borders of the round proliferating (RP), columnar proliferating (CP) and pre-hypertrophic/hypertrophic (P-H/H) zones. (L,M) Quantification of the length of the growth plate (L) and length of each growth plate zone relative to the total length of the growth plate (M) in *n*=3 control (*Col2a1cre^−ve^*) and *n*=4 *nls-YAP5SA^KI^*^/*+*^*Col2a1cre^+ve^* E17.5 pups. Data were analysed and found to be significant by unpaired *t*-test (L) and two-way ANOVA with growth plate zone and genotype as the independent variables, relative zone size as the dependent variable (M). The effect of genotype on relative zone size was not significant (*P*=0.9823). (N) Immunostaining for Ki67 in the zones of the proximal growth plate of the tibia. Data are representative of *n*=3 control (*Col2a1cre^−ve^*) and *n*=4 *nls-YAP5SA^KI^*^/*+*^*Col2a1cre^+ve^* E17.5 pups. (O,P) Quantification of proliferating cells (O) and cell density (P) of samples from F. Data were analysed by two-way ANOVA with growth plate zone and genotype as the independent variables, percentage proliferating cells (O) or cell number (P) as the dependent variable. The effect of genotype on proliferation (O) was not significant (*P*=0.6278) and effect of genotype on cell density (P) was significant (*P*=0.0049). Scale bars: 3 mm (A-J); 200 µm (K, upper); 170 µm (K, lower); 50 µm (N).

Two previous studies found that moderately increased YAP activity in chondrocytes either during embryonic development (Deng et al., 2016) or postnatal development (Goto et al., 2018) led to a proportional decrease in body size. Similarly, we found a decrease in body size when we expressed the nuclear-targeted *YAP* allele, *nls-YAP5SA*, in chondrocytes (*nls-YAP5SA^KI^*^/*+*^*Col2a1cre^+ve^*); however, this phenotype was substantially more severe than the previously reported phenotypes and was accompanied by catastrophic chondrodysplasia resembling achondrogenesis (Fig. 5A-F). Skeletal preparations revealed extremely dysmorphic skeletal elements throughout the body, including highly dysplastic facial bones, ectopic bone elements alone the spine and abnormal rib cage and limbs (Fig. 5G-J). As the limbs appeared less affected relative to the other skeletal elements, we examined the proximal growth plate of the tibia histologically (Fig. 5K). The growth plate in the *nls-YAP5SA^KI^*^/*+*^*Col2a1cre^+ve^* tibia was ∼30% smaller than the control and the total Alcian Blue-stained area was similarly reduced, though the relative size of each chondrocyte growth zone was unchanged (Fig. 5K-M). Proliferation was assessed by Ki67 staining (Fig. 5N) and no change was observed in any chondrocyte zone (Fig. 5O), once again in contrast to the increase in proliferation observed in primary chondrocytes *in vitro* (Fig. 1C,D). Apoptosis was also unchanged (Fig. S3C). However, cell density was increased in *YAP5SA^KI^*^/*+*^*Col2a1cre^+ve^* samples (Fig. 5P), suggesting decreased ECM in the intracellular spaces, mirroring the decreased cell density and increased percentage of area occupied by cartilage matrix in *Yap^fl^*^/*fl*^*Taz^fl^*^/*fl*^*Col2a1cre^+ve^* tibias (Fig. 4E,L). These results show that expression of a nuclear-targeted YAP protein in chondrocytes *in vivo* does not affect cell proliferation and instead causes severely abnormal cartilage morphogenesis and decreased size of skeletal elements, possibly via altering the ECM.

**Fig. 6.**
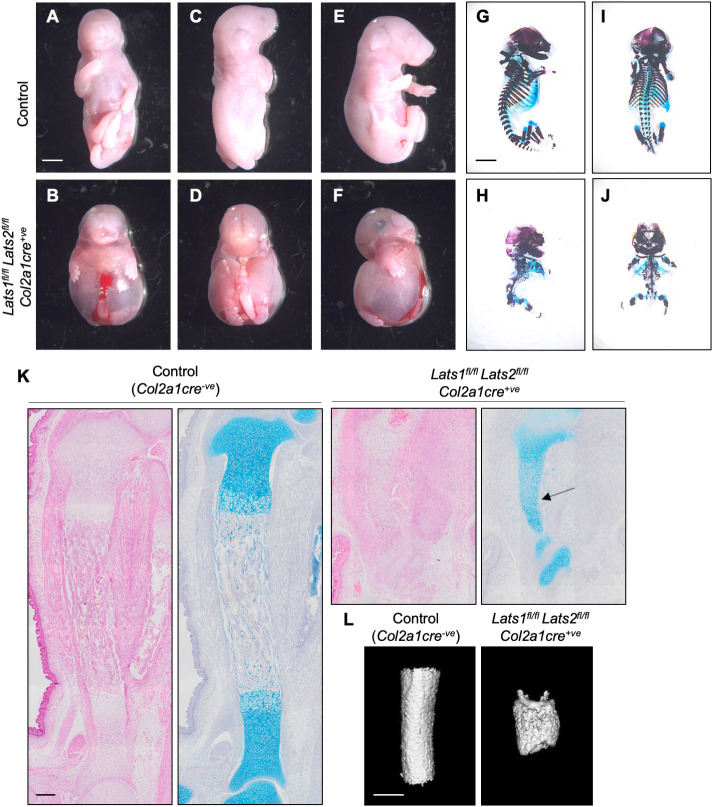
**Severe chondrodysplasia in *Lats1*/*2* chondrocyte-specific knockout pups.** (A-F) Ventral (A,B), dorsal (C,D) and lateral (E,F) views of control (*Col2a1cre^−ve^*) (A,C,E) and *Lats1*/*2^fl^*^/*fl*^*Col2a1cre^+ve^* (B,D,F) E18.5 pups. (G-J) Lateral (G,H) and dorsal (I,J) views of skeletal preparations of control (*Lats1^fl^*^/*+*^*Lats2^fl^*^/*fl*^*Col2a1cre^+ve^*) (G,I) and *Lats1*/*2^fl^*^/*fl*^*Col2a1cre^+ve^* (H,J) E17.5 pups. (K) H&E- (left image per genotype) and Alcian Blue- (right image per genotype) stained histological sections of the proximal growth plate of the tibia. Data are representative of *n*=3 control (*Col2a1cre^−ve^*) and *n*=3 *Lats1*/*2^fl^*^/*fl*^*Col2a1cre^+ve^* E17.5 pups. (L) Measurements of femurs and tibias from microCT analysis of *n*=3 control (*Col2a1cre^−ve^*) and *n*=3 *Yap^fl^*^/*fl*^*Taz^fl^*^/*fl*^*Col2a1cre^+ve^* pups. Scale bars: 3 mm (A-J); 200 µm (K); 400 μm (L).

To confirm the biological relevance of the *nls-YAP5SA^KI^*^/*+*^*Col2a1cre^+ve^* overexpression phenotype, we inactivated endogenous Hippo signalling through cartilage-specific conditional knockout of the upstream negative regulators of YAP, *Lats1* and *Lats2* (*Lats1*/*2^fl^*^/*fl*^*Col2a1cre^+ve^*). At E18.5, compared with *Col2a1cre^−ve^* control littermates, *Lats1*/*2^fl^*^/*fl*^*Col2a1cre^+ve^* pups once again had extremely severe chondrodysplasia that was highly similar to and more severe than that of *nls-YAP5SA^KI^*^/*+*^*Col2a1cre^+ve^* mutants (Fig. 6A-J). Staining of tibias with Alcian Blue revealed that the *Lats1*/*2^fl^*^/*fl*^*Col2a1cre^+ve^* cartilaginous structure was strongly decreased in total area and intensity of staining compared with control tibias (Fig. 6K). However, a core of chondrocytes persisted throughout the length of the tibia (arrow in Fig. 6K), indicative of a failure of chondrocytes to completely differentiate and clear, through either apoptosis or transdifferention into osteoblasts (Yang et al., 2014; Zhou et al., 2014). Micro CT scanning of the tibias revealed the presence of mineralised bone surrounding a persistent shaft of chondrocytes, albeit appearing non-uniform in its surface (Fig. 6L). These results confirm that the *nls-YAP5SA^KI^*^/*+*^*Col2a1cre^+ve^* mutants are biologically relevant and demonstrate that suppression of YAP/TAZ activity in cartilage by the Hippo pathway is crucial for cartilage morphogenesis and normal skeletal development.

### Modulation of YAP/TAZ affects expression of matrix remodelling genes *in vivo*

Conflicting reports from previous studies concluded that YAP activity in chondrocytes functions primarily to either repress chondrocyte differentiation, by promoting *Sox6* expression and repressing *Col10a1* (Deng et al., 2016; Goto et al., 2018) or to promote chondrocyte differentiation by repressing *Sox9* (Deng et al., 2016; Goto et al., 2018). We therefore sought to re-examine the expression of COL10a1 (COLX) and SOX9 in tibial growth plates from our YAP/TAZ loss-of-function and gain-of-function animals. We saw an expansion of the COLX-staining domain in *Yap^fl^*^/*fl*^*Taz^fl^*^/*fl*^*Col2a1cre^+ve^* but only a slight reduction in domain size in *YAP5SA^KI^*^/*+*^*Col2a1cre^+ve^* tibias (Fig. S5A-D), suggesting that the expansion of the COLX-stained hypertrophic zone may reflect a delay in differentiation rather than an effect of loss of direct regulation by YAP/TAZ. Strikingly, we found that the pattern and levels of expression of SOX9 was essentially normal in *Yap^fl^*^/*fl*^*Taz^fl^*^/*fl*^*Col2a1cre^+ve^* tibias and in *nls-YAP5SA^KI^*^/*+*^*Col2a1cre^+ve^* tibias relative to total growth plate size (Fig. S5A-D). These results argue against an essential function for YAP/TAZ in regulating chondrocyte cell differentiation via direct repression of *Sox9* or *Col10a1* gene expression. Nevertheless, we were able to detect a mild reduction of SOX9- and, to a much lesser extent, COLX-stained area in our *Lats1*/*2^fl^*^/*fl*^*Col2a1cre^+ve^* tibial growth plates (Fig. S5E), similar to that reported for cartilage-specific knockout of *Mob1a*/*b* (Goto et al., 2018) – but these effects may be a secondary consequence of the highly abnormal morphology of these growth plates.

**Fig. 7.**
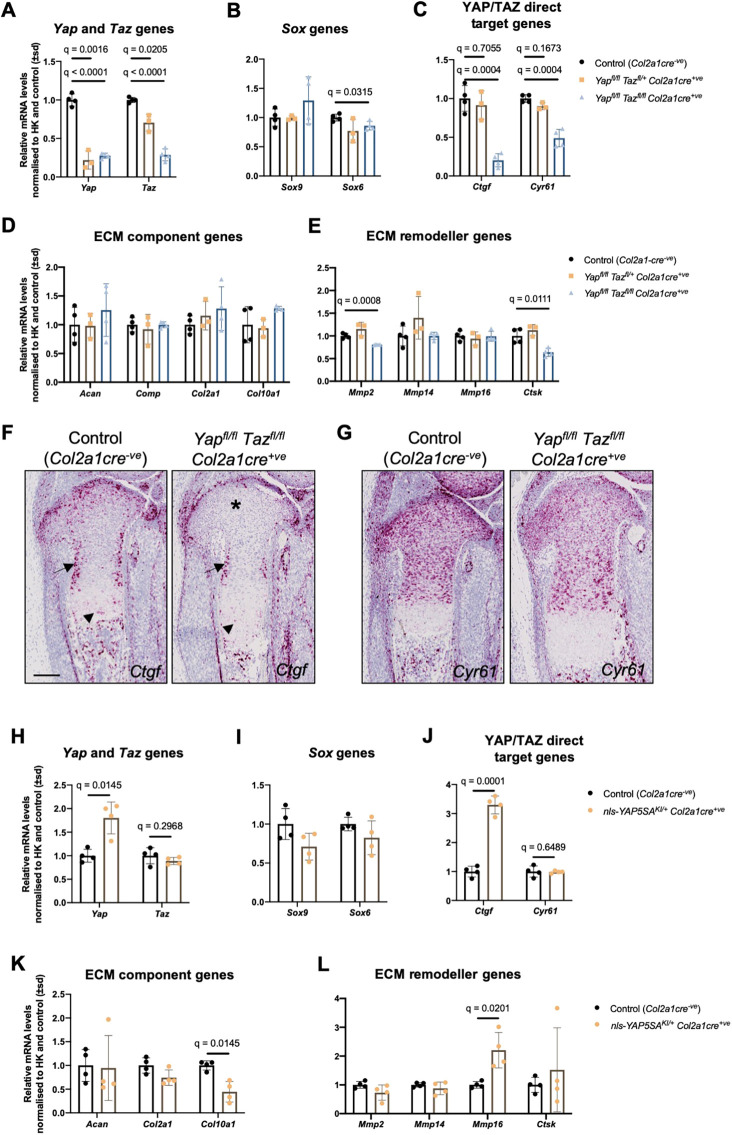
**YAP/TAZ modulation affects expression of ECM remodeller genes.** (A-E) RTqPCR analysis of laser-microdissected tibial growth plates of four E17.5 *Yap^fl^*^/*fl*^*Taz^fl^*^/*fl*^*Col2a1cre^+ve^* mutants compared with four control (*Col2a1cre^−ve^*) and three *Yap^fl^*^/*fl*^*Taz^fl^*^/*+*^*Col2a1cre^+ve^* littermates. *Yap* and *Taz* (A) and their known target genes *Ctgf* and *Cyr61* (C) were analysed, along with selected Sox genes (B) and genes encoding cartilage ECM components (D) and remodellers (E). Data were analysed by *t*-tests comparing each mutant with control and adjusted for multiple testing with a 5% false discovery rate. Discoveries are given as *q* values. The absence of a *q* value indicates *q* was not statistically significant (*q*>0.05). (F,G) RNA *in situ* hybridisation to known YAP/TAZ target genes *Ctgf* (F) and *Cyr61* (G). *Ctgf* displays a substantial reduction of signal in the tibial growth plate in the absence of *Yap*/*Taz* (asterisk in F), though some expression persists adjacent to the perichondrium (arrows in F) and moderate reduction in signal is observed in the hypertrophic zone (arrowheads in F). *Cyr61* signal is generally reduced in *Yap*/*Taz* mutant growth plates compared with controls (G). *n*=4 control (*Col2a1cre^−ve^*) and *n*=4 *Yap^fl^*^/*fl*^*Taz^fl^*^/*fl*^*Col2a1cre^+ve^* tibial growth plates. (H-L) RTqPCR analysis of laser-microdissected tibial growth plates of four E17.5 control (*Col2a1cre^−ve^*) and four *nls-YAP5SA^KI^*^/*+*^*Col2a1cre^+ve^* littermates. *Yap* and *Taz* (H) and their known target genes *Ctgf* and *Cyr61* (J) were analysed, along with selected Sox genes (I) and genes encoding cartilage ECM components (K) and remodellers (L). Data were analysed by multiple *t*-tests and adjusted for multiple testing with a 5% false discovery rate. Discoveries are given as *q* values. The absence of a *q* value indicates *q* was not statistically significant (*q*≥0.05). Scale bar: 100 µm (F,G).

Descriptions of molecular functions of YAP and TAZ in chondrocytes have been derived predominantly from *in vitro* analyses (Deng et al., 2016; Goto et al., 2018). However, we describe here a significant departure in phenotypic outcomes *in vitro* compared with *in vivo*. Therefore, to more closely quantify the molecular consequences of YAP/TAZ modulation *in vivo*, we performed RTqPCR on laser microdissected tibial growth plate sections. Compared with controls (*Col2a1cre^−ve^*), in *Yap^fl^*^/*fl*^*Taz^fl^*^/*fl*^*Col2a1cre^+ve^* growth plates, *Yap* and *Taz* expression was reduced by 75% (Fig. 7A). Contrary to the previous findings that YAP/TAZ represses *Sox9* or induces *Sox6* (Deng et al., 2016; Goto et al., 2018), we did not detect a substantial change in either *Sox9* expression or *Sox6* levels in the double knockout growth plates (Fig. 7B).

The skeletal defects observed here in *Yap^fl^*^/*fl*^*Taz^fl^*^/*fl*^*Col2a1cre^+ve^* mutants are highly reminiscent of the skeletal phenotypes observed in mice carrying mutations in *Ctgf*, a known direct target of YAP/TAZ (Zhang et al., 2009; Zhao et al., 2008), including bowed tibia and ribs, unelevated cleft palate, abnormal Meckel's cartilage and increased ECM in the growth plate with an elongated pre-hypertrophic/hypertrophic zone (Ivkovic et al., 2003). We therefore examined expression of *Ctgf* and the closely related *Cyr61*, also a target of YAP/TAZ (Lai et al., 2011), and found a substantial reduction in expression of both genes in *Yap^fl^*^/*fl*^*Taz^fl^*^/*fl*^*Col2a1cre^+ve^* samples compared to controls (Fig. 7C). Strikingly, neither gene showed any decrease in the growth plates from *Yap^fl^*^/*fl*^*Taz^fl^*^/*+*^*Col2a1cre^+ve^* pups, which do not display any skeletal or lethal phenotypes, suggesting that dysregulation of *Ctgf*, perhaps with some contribution from a reduction in *Cyr61* levels, underlies the cartilage defects observed in *Yap^fl^*^/*fl*^*Taz^fl^*^/*fl*^*Col2a1cre^+ve^* mutants.

To explore the molecular basis for the increased amount of ECM in the *Yap^fl^*^/*fl*^*Taz^fl^*^/*fl*^*Col2a1cre^+ve^* mutants, we next examined the expression of genes encoding major cartilage ECM components, namely aggrecan (*Acan*), cartilage oligomeric matrix protein (*Comp*), *Col2a1* and *Col10a1*. We did not detect any significant change in expression of these genes (Fig. 7D), indicating that the increase in ECM area is not due to increased production of the matrix proteins. The differentiation of chondrocytes and the remodelling of cartilage into mineralised bone requires the activity of numerous proteases, including matrix metalloproteases (MMPs) and Cathepsin K (CTSK) and a decrease in protease activity can reduce the turnover of ECM proteins. Though we could not reliably detect transcripts of *Mmp9* or *Mmp13* – two key protease regulators of cartilage remodelling (Stickens et al., 2004; Vu et al., 1998) – we saw a reduction in *Ctsk* and *Mmp2* expression in *Yap^fl^*^/*fl*^*Taz^fl^*^/*fl*^*Col2a1cre^+ve^*, but not *Yap^fl^*^/*fl*^*Taz^fl^*^/*+*^*Col2a1cre^+ve^*, mutant samples (Fig. 7E). CTSK cleaves the major cartilage ECM components collagen II and aggrecan (Hou et al., 2003; Kafienah et al., 1998), suggesting the increased area of ECM in double homozygous mutants may represent accumulated ECM proteins owing to decreased rate of degradation by CTSK, MMP2 and perhaps other ECM proteases.

We next confirmed the RTqPCR reduction in *Ctgf* and *Cyr61* by *in situ* hybridisation and saw comparable reductions in signal (Fig. 7F,G). Unexpectedly, we did not observe a strong signal for *Ctgf* in its published predominant expression domain, namely the pre-hypertrophic/hypertrophic zone (Ivkovic et al., 2003); however, this may be a histological artefact of the *in situ* protocol which does not preserve cellular material of this region well.

To examine the molecular consequence of YAP hyperactivation, we performed RTqPCR analysis on microdissected tibial sections from control (*Col2a1cre^−ve^*) and *YAP5SA^KI^*^/*+*^*Col2a1cre^+ve^* E17.5 samples. We confirmed increased *Yap* levels to almost twice that of control and no change in *Taz* expression (Fig. 7H). Here, we saw no change to *Sox9* or *Sox6* levels (Fig. 7I). In contrast, *Ctgf* levels were up 3-fold in *YAP5SA^KI^*^/*+*^*Col2a1cre^+ve^* growth plates, mirroring the decrease we observed in *Yap^fl^*^/*fl*^*Taz^fl^*^/*fl*^*Col2a1cre^+ve^* samples, although interestingly *Cyr61* was unchanged (Fig. 7J). Of the ECM component genes examined, *Col10a1* was significantly reduced (Fig. 7K) and though neither *Mmp2* nor *Ctsk* showed a significant change in *YAP5SA^KI^*^/*+*^*Col2a1cre^+ve^* samples, there was a substantial increase in *Mmp16* expression (Fig. 7L). MMP16 is a membrane-tethered MMP capable of degrading type II collagen, and dual deletion of *Mmp16* and the closely related *Mmp14* in mice results in increased ECM accumulation in the femoral growth plate (Shi et al., 2008). This implies a reciprocal mechanism in our YAP/TAZ loss- and gain-of function mutants with regards ECM remodelling in the cartilage through modulation of CTGF and CYR61, as well as various matrix remodelling enzymes.

Thus, our findings refine the current model, namely that YAP and TAZ control cartilage development exclusively via direct regulation of *Sox9*, and support the notion that regulation of cartilage morphogenesis, particularly by remodelling the ECM through regulation of *Ctgf*, *Cyr61* and various ECM proteases, is a primary physiological function for Hippo-YAP/TAZ signalling in this tissue.

## DISCUSSION

In this study, we have identified a striking discrepancy between *in vitro* and *in vivo* functions of the Hippo-YAP/TAZ pathway in chondrocytes. Although primary cultured chondrocytes *in vitro* responded to YAP/TAZ loss-of-function or YAP gain-of-function according to the canonical understanding of YAP/TAZ being positive regulators of cell proliferation, identical genetic alterations *in vivo* did not affect chondrocyte proliferation. Further characterisation of the phenotypes of these animals revealed that YAP/TAZ are also not essential to regulate chondrocyte cell differentiation *in vivo*, and instead function primarily to control cartilage morphogenesis, including via regulation of the ECM. Our findings have medical relevance because ∼40% of patients in a family carrying a loss-of-function mutation in the *YAP1* gene have been reported to have cleft palate/lip/uvula at birth (Williamson et al., 2014) and our results indicate that these mutations may be causative, as our mouse *Yap*/*Taz* knockouts produce strongly penetrant cleft palate and complete neonatal lethality. Furthermore, skeletal malformations, including achondrogenesis and chondrodysplasia, are common but still poorly understood birth defects in humans (Feldkamp et al., 2017; Swarr and Reid Sutton, 2010) and our results implicate loss of Hippo pathway signalling as potentially causative, as the mouse *Lats1*/*2* knockouts and *Yap* constitutively active mutants exhibit a catastrophically malformed cartilage and skeleton at the end of gestation.

Our findings help resolve the conflicting reports for Hippo pathway function in regulating chondrocyte cell proliferation. YAP/TAZ have been reported widely as positive regulators of cell proliferation in chondrocytes *in vitro* (Deng et al., 2016; Yang et al., 2016; Zhong et al., 2013) and in the mouse prechondrocytic cell line ATDC5 *in vitro* (Yang et al., 2017). In one exception, Goto et al. report that activation of YAP/TAZ by depletion of *MOB1a*/*b* in the human chondrosarcoma cell line H-EMC-SS resulted in a reduction of proliferation (Goto et al., 2018). This *in vitro* observation agrees with the reduced proliferation they observed in the growth plates of the shortened limbs of adult mice with neonatal-deletion of *Mob1a*/*b*, but is in contrast with the study by Deng et al., which described increased proliferation in YAP transgenic-overexpressing late-gestation pups, despite having a substantially smaller overall body size (Deng et al., 2016). We find no change in cell proliferation *in vivo* upon modulation of YAP/TAZ activity in *Yap^fl^*^/*fl*^*Taz^fl^*^/*fl*^*Col2a1cre^+ve^* pups or in *nls-YAP5SA^KI^*^/*+*^*Col2a1cre^+ve^* mutants compared with controls and we instead observe changes in cell density and the ECM *in vivo*. Thus, although chondrocytes in culture depend strongly on YAP/TAZ for proliferation (which could reflect a programme of tissue regeneration after damage), chondrocytes *in vivo* employ Hippo-YAP/TAZ signalling primarily to regulate morphogenesis during development.

The two known direct targets of YAP/TAZ, *Ctgf* and *Cyr61*, have well-documented roles in cartilage development (O'Brien and Lau, 1992; Wong et al., 1997). CTGF has been shown to bind to ECM components including aggrecan (Aoyama et al., 2009) as well as to cell surface integrins (Nishida et al., 2007) and a range of growth factors. The defects observed here in *Yap^fl^*^/*fl*^*Taz^fl^*^/*fl*^*Col2a1cre^+ve^* mutant animals closely phenocopy the defects described in *Ctgf* knockout animals, including malformed Meckel's cartilage and cleft palate (Ivkovic et al., 2003). The decrease in *Ctgf* mRNA levels to ∼25% of control suggests that loss of *Ctgf* is a major contributor to the *Yap^fl^*^/*fl*^*Taz^fl^*^/*fl*^*Col2a1cre^+ve^* skeletal defects. Interestingly, 2-fold overexpression of *Ctgf* resulted in an overall larger body size of animals (Tomita et al., 2013), suggesting that increased *Ctgf* expression in our *nls-YAP5SA^KI^*^/*+*^*Col2a1cre^+ve^* mutants is not the only YAP target affected here to cause the observed catastrophic chondrodysplasia in those mutants. Overexpression of *Cyr61* in chondrocytes leads to chondrodysplasia (Akiyama et al., 2004; Zhang et al., 2016). However, we did not detect an increase in *Cyr61* expression in our *nls-YAP5SA^KI^*^/*+*^*Col2a1cre^+ve^* mutant animals, once again supporting the view that *Ctgf* and *Cyr61* are important YAP/TAZ targets in cartilage, yet there may be additional target genes that contribute to the phenotypes observed.

A previous report concluded that a major function of YAP/TAZ in cartilage development is to inhibit chondrocyte differentiation by direct transcriptional repression of the *Sox9* gene (Deng et al., 2016; Goto et al., 2018). In contrast, in chondrocytes *in vitro*, *Sox9* mRNA levels have been reported to be both positively (Li et al., 2018) or negatively (Karystinou et al., 2015; Yang et al., 2017) regulated by YAP. The phenotype of the *nls-YAP5SA* overexpression mutant reported here bears some resemblance to the published phenotype of *Sox9^fl^*^/*fl*^*Col2a1Cre^+ve^* pups (Akiyama et al., 2002) and to animals with chondrocyte-specific expression of constitutively active β-catenin (Akiyama et al., 2004). However, our results argue against an essential and physiological role for YAP/TAZ in directly regulating *Sox9* gene expression during development, as neither our *Yap^fl^*^/*fl*^*Taz^fl^*^/*fl*^*Col2a1cre^+ve^* double knockout or *nls-YAP5SA^KI^*^/*+*^*Col2a1cre^+ve^* overexpressing animals exhibited a change of *Sox9* expression by RTqPCR (Fig. 7B,I). Furthermore, SOX9 immunostaining in tibial growth plates of these mutants was not changed relative to the overall smaller size of the growth plate (Fig. S5A-D). Thus, the primary physiological function of YAP/TAZ during embryonic cartilage development is not to regulate chondrocyte differentiation via *Sox9* modulation, but rather to direct cartilage morphogenesis through regulation of *Ctgf* and *Cy61* as well as other target genes. The reduced *Sox9* expression in our *Lats1*/*2^fl^*^/*fl*^*Col2a1cre^+ve^* mutants may reflect an extreme scenario in which both YAP and TAZ are strongly activated, possibly relevant to the regenerative response, rather than normal development (Fig. S5E).

Given that chondrocytes secrete large amounts of specialised collagen II- and aggrecan-based ECM material to produce cartilage, it is plausible that the Hippo pathway functions to sense and regulate ECM synthesis and/or remodelling. This concept is consistent with the known function of integrins in binding to the ECM and transducing signals via Hippo-YAP/TAZ in other tissues such as skin (Elbediwy et al., 2016), pancreas (Mamidi et al., 2018), tooth (Hu et al., 2017), blood vessels (Wang et al., 2016b), as well as in mesenchymal stem cells (Sabra et al., 2017; Tang et al., 2013), osteoblasts (Kaneko et al., 2014) and cancer cells (Kim and Gumbiner, 2015; Wong et al., 2016). Interestingly, matrix metalloprotease enzymes are downstream target genes of YAP that can be induced upon integrin binding to stiff matrix substrates (Chakraborty et al., 2017; Nukuda et al., 2015) or dense matrix (Stanton et al., 2019). Our results show that YAP/TAZ can positively regulate expression of *Ctsk, Mmp2* and *Mmp16* in addition to *Ctgf* and *Cyr61.* In particular, induction of *Mmp16* along with *Ctgf* in the *YAP5SA^KI^*^/*+*^*Col2a1cre^+ve^* samples could contribute to the chondrodysplasia phenotype observed. Consistent with our findings, a recent study has suggested that YAP/TAZ mediates TGF-β-induction of bone matrix remodelling factors *Ctsk*, *Mmp13* and *Mmp14* (Kegelman et al., 2020). Thus, chondrocytes may also employ the Hippo-YAP/TAZ pathway to sense mechanical forces acting via the ECM and regulate the developmental remodelling response via *Ctgf*, *Cyr61* and several matrix remodelling enzymes. In support of this notion, a recent study has demonstrated that YAP localisation and activity in the embryonic cartilaginous humerus is depended on mechanical stimulation derived from the surrounding muscle (Shea et al., 2020). Our findings also have important medical relevance, further implicating the Hippo-YAP/TAZ pathway in human birth defects including chondrodysplasia and cleft palate (Williamson et al., 2014).

## MATERIALS AND METHODS

### Mice

All animal (*Mus musculus*) experiments were carried out in accordance with the United Kingdom Animal Scientific Procedures Act (1986) and UK Home Office regulations under project license numbers 70/7926 and PDCC6E810. The *Yap^fl^* and *Taz^fl^* (*Wwtr1fl*) (Gruber et al., 2016), *nls-YAP5SA^KI^* (Vincent-Mistiaen et al., 2018), *Lats^fl^* and *Lats2^fl^* (Yi et al., 2016), *Col2a1cre* (Ovchinnikov et al., 2000) and *Col2a1cre-ERT* (Nakamura et al., 2006) strains have all been previously described. For timed matings, E0.5 was designated as midday following the morning of finding the vaginal plug. All mice were maintained on a mixed, predominantly C57Bl/6J background.

### Primary chondrocyte culture

Primary chondrocytes from the ribs and sterna were isolated essentially as described previously (Mirando, 2014). Briefly, the ribs and sterna of P0 for wild-type compared with *Col2a1cre^+ve^* pups or E17.5 pups for all other genotypes were dissected and excess overlying musculature removed before incubation in 2 mg/ml Pronase (Sigma-Aldrich) for 45 min at 37°C with agitation then 3 mg/ml Collagenase D (Roche) for 45 min at 37°C following extensive rinsing with PBS. After additional rinses with PBS to clear remaining soft tissue, cartilage elements were incubated again in 3 mg/ml Collagenase D for 3-6 h until a single-cell suspension was achieved, with regular gentle titration with wide-bore 1 ml pipette tips. Cells were filtered then plated at 3000 cells per well of a black polystyrene flat-bottom (with micro-clear bottom) 96-well plate (Greiner) in DMEM supplemented with 10% fetal calf serum and pen-strep. For the *nls-YAP5SA^KI^*^/*+*^*Col2a1cre-ERT^+ve^* experiment, cells were treated 24 h after plating with 1 μM 4-hydroxytamoxifen in ethanol or ethanol only. Confluence was measured by automated detection of cell confluence in 3 h serial photographs on an Essen IncuCyte. Apoptosis was assessed by the inclusion of apoptosis marker NucView488 (Biotium).

### Skeletal preparations and high resolution episcopic microscopy

Skeletal preparations were performed as previously described (Rigueur and Lyons, 2014). Samples were prepared for HREM by incubation in Bouin's fixation for a minimum of 3 days followed by extensive washing in PBS and dehydration, before incubation in JB-4/Dye mix for up to 4 weeks to ensure proper sample penetration and then embedded and imaged as previously described (Mohun and Weninger, 2012; Weninger et al., 2018) (https://dmdd.org.uk/hrem/). Meckel's cartilages were isolated from 3D reconstructed images using automated thresholded extraction in Analyze v12.0 visualisation and analysis software (AnalyzeDirect).

### Histological analysis and *in situ* hybridisation

Whole E17.5 pups were fixed in 10% neutral buffered formalin for two days then transferred to 70% ethanol. Rear limbs were dissected and processed by standard histological methods to generate 3 μm paraffin sections, which were stained with H&E or immunostained with DAB detection for anti-Ki67 (1:350; AB16667, Abcam), anti-YAP (Santa Cruz Biotechnology, sc-101199; 1:200 IF, 1:1000 IB) or anti-TAZ (anti-WWTR1, 1:100; HPA007415, Atlas Antibodies) using standard protocols. The specificity of the YAP and TAZ antibodies was previously confirmed in knockout skin (Elbediwy et al., 2016). Growth plate zones were defined based on H&E morphology except for the pre-hypertrophic and hypertrophic zones, which were combined and considered as a single pre-hypertrophic/hypertrophic zone. For Ki67^+ve^ cell counting, the proximal growth plate of the tibia was divided into the round proliferating, columnar proliferating and pre-hypertrophic/hypertrophic zones and a box of consistent size (for each zone) drawn within the centremost region of each defined zone. One tibial growth plate section was examined per biological replicate. Ki67 immuno-positive and -negative cells were counted manually and the number of Ki67 immuno-positive cells expressed as the percentage total. Cells in the pre-hypertrophic/hypertrophic zone were negative for Ki67 staining. For cell counting per zone for Fig. 4E, the cells counted in the columnar proliferating zone were divided by an ‘area factor’ representing the area of counted cells in the columnar proliferating zone divided by the area of counted cells in the round or pre-hypertrophic/hypertrophic zones, to account for the difference in area counted between the central zone compared with the other two zones. For DAPI-stained cell counts, see ‘EdU proliferation’ section below.

For fluorescent immunostaining, paraffin sections (anti-SOX9, anti-COLXa1) were taken, tibias were dewaxed and then underwent a citrate buffer (pH 6.0) retrieval step. Fresh-frozen cryosections (anti-YAP) were fixed in 4% paraformaldehyde for 10 min direct from storage then washed in PBS. All samples were then permeabilised for 10 min in PBS plus 0.1% Tween-20 then blocked for an hour with 5% normal goat serum in PBS plus 0.1% Triton X-100. Primary antibodies were diluted in 1% bovine serum albumin (BSA) in PBS at 1:250 for anti-SOX9 (AB5535, Sigma-Aldrich), 1:50 for anti-COLXaI (14-9771-82, Invitrogen) and 1:100 for anti-YAP (14074, Cell Signaling Technology). Alexa Fluor 488 or 564 goat secondary antibodies against the appropriate species were diluted at 1:200 in 1% BSA in PBS and DAPI was used to stain nuclei.

TUNEL staining was performed on sectioned E17.5 tibia using the Click-iT™ Plus TUNEL Assay kit (Thermo Fisher Scientific) following the manufacturer's protocol. For RNA *in situ* hybridisation, RNAscope 2.5 HD Manual Assay (ACDBio) was performed according to manufacturer's instructions, using RNAscope probes for *Ctgf* (314541, ACDBio) and *Cyr61* (429001, ACDBio).

### Micro CT

*Ex vivo* CT scans were acquired using a SkyScan 1176 CT scanner (Bruker MicroCT) with the source voltage set to 50 kV, the source current set to 500 µA, a frame averaging of 5, and a 0.5° step size over a 360° trajectory. The scans were reconstructed using NRecon v1.7.3.0 software (Bruker MicroCT) with an 8.57 µm isotropic voxel size and analysed using Analyze v12.0 visualisation and analysis software (AnalyzeDirect).

### Quantification of ECM quantification

Dewaxed slides were incubated in Alcian Blue solution (1% Alcian Blue in 3% acetic acid) for 5 min before being washed extensively under running water and counterstained. To calculate percentage coverage by ECM, images were filtered in ImageJ with a Gaussian blur, then colours split and the red channel retained. Growth plate regions were isolated from the centre of each chondrocyte zone and threshold using default settings. Percentage thresholded area was then measured.

### EdU proliferation

Pregnant dams were injected with 30 mg/kg EdU in PBS 2 h before collection at E17.5. Pups were processed as described above for histological analysis. To assess proliferation, samples were processed for EdU staining with the Click-iT EdU Alexa Fluor 488 Imaging Kit (Invitrogen) according to a published protocol (Mead and Lefebvre, 2014). Images were acquired for nuclei (Hoechst) and EdU staining and a box of consistent size was drawn in the centremost region of each chondrocyte compartment. One tibial growth plate section was examined per biological replicate. EdU-positive and DAPI-stained nuclei were automatically counted using CellProfiler (McQuin et al., 2018). DAPI staining was used to count cells along length of growth plate: an area of consistent width down the centre length of the growth plate was isolated. Images were processed in ImageJ by applying Gaussian blur filter followed by Huang thresholding and watershedding. The number and position of particles were detected, excluding particles with an area of less than 100 pixels, and the total number of particles or the longitudinal distance along the *y*-axis of the image was collected. Positional information was then binned in Prism 8.

### Laser capture microdissection, RNA extraction and RTqPCR analysis

E17.5 lower limbs were fresh frozen into Optimal Cutting Temperature reagent, sectioned at 10 µm and 8-12 sections collected onto UV-treated PEN-Membrane 4.0 µm slides (Leica). Directly before laser capture, slides were removed from storage at −80°C and washed twice in ice-cold 95% EtOH for 2 min. After airdrying the slides at room temperature, sections were visualised in brightfield on the Laser Capture Microdissector (LMD7000, Leica) and a 300 µm-wide box drawn down the midline on the tibial growth plate. Then 7-12 growth plate regions per slide (i.e. per biological replicate) were dissected and collected into 0.5 ml tubes, and 200 µl of TRIzol Reagent (Invitrogen) was added to sections before storage at −20°C. RNA was extracted using the Trizol manufacturer's instructions. cDNA was synthesised from isolated RNA using the Maxima cDNA Synthesis Kit (Thermo Fisher Scientific). RTqPCR was performed on the QuantSudio 7 Flex PCR System (Applied Biosystems) using PowerUp SYBR Green Master Mix (Thermo Fisher Scientific). Primers are listed in Table S1.

### Statistical methods

The linear growth phase of primary chondrocytes was analysed by linear mixed models, including a fixed effect for elapsed time and a random effect for each technical repeat to account for random variance in experimental factors. The models were fitted in R using the function lmer of the R package lme4 (Bates et al., 2015). The significance of the genotype was calculated by comparing the variance of the fitted models with and without the fixed genotype effect using an F-test with Satterthwaite's approximation for degrees of freedom (Kuznetsova et al., 2017). All remaining statistical analyses were performed in Prism 8.

## Supplementary Material

Supplementary information

Reviewer comments
