# Supplementary material for: Control of skeletal morphogenesis by the Hippo-YAP/TAZ pathway
Source: Development. 2020 Nov 12;147(21):dev187187. doi: 10.1242/dev.187187 (PMC7673359; doi:10.1242/dev.187187)
Supplement: Supplementary information [file develop-147-187187-s1.pdf]

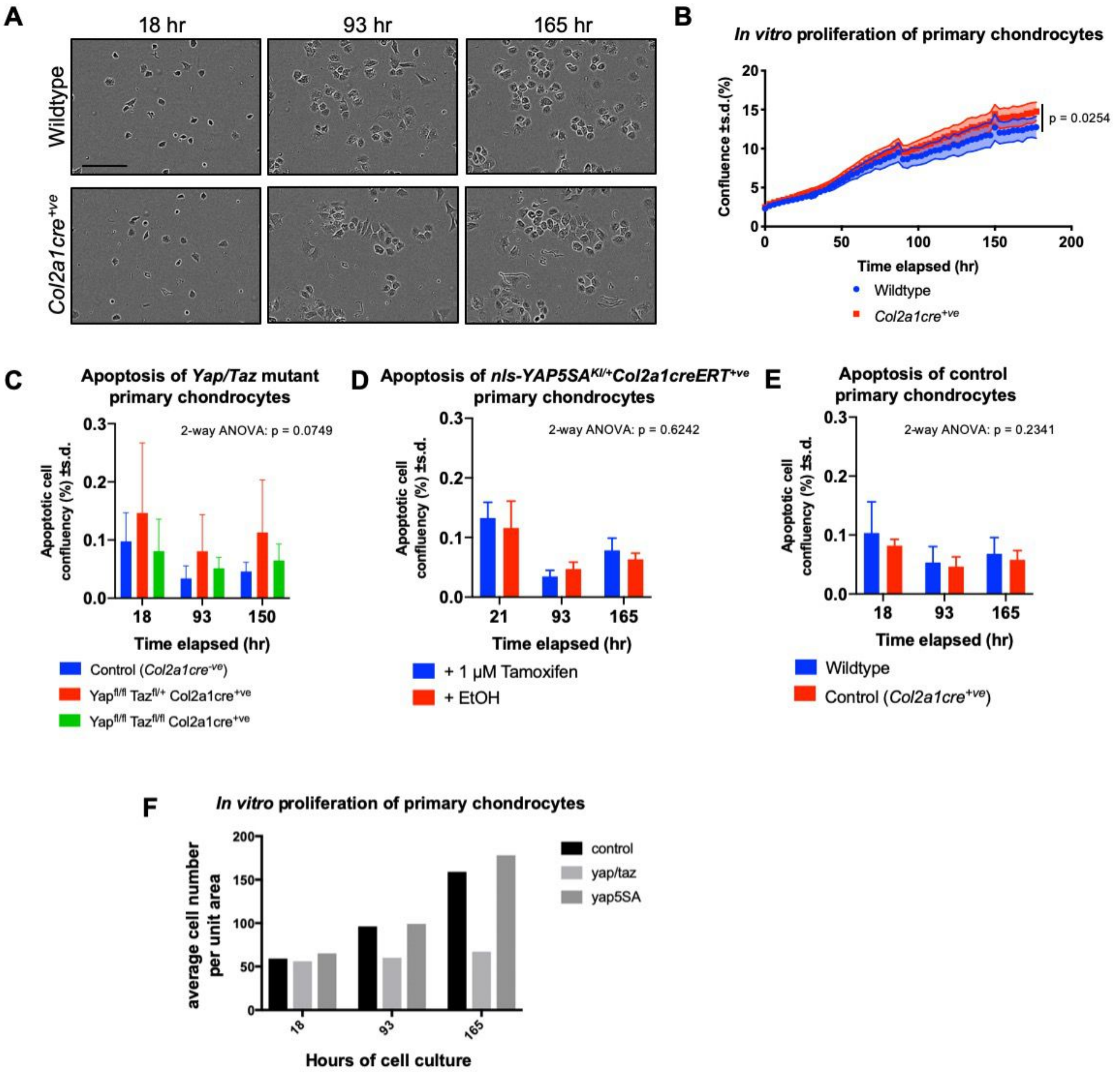

Figure S1. YAP/TAZ are required for primary chondrocyte proliferation *in vitro*, but are not required to prevent apoptosis.

A) Primary chondrocyte cultures from ribcages and sterna of wild-type or *Col2a1cre^{+ve}* P0 pups plated at low density (3000 cells per well).

B) Proliferation, measured by confluence (percentage cell coverage) of field of view, of cultures from A). Data represent average of individual pups (biological replicates), the averages of which were derived from 6 wells (technical replicates). *n* = 3 wild-type and 5 *Col2a1cre^{+ve}* P0 pups. Linear growth phase was measured by linear mixed model.

C-E) Levels of apoptosis, measured by confluence (percentage cell coverage) of field of view of apoptotic cells stained with NucView488 as a percentage of total cell confluence. Data represent C) 9 technical replicates per indicated genotype; D) 3 *nls-YAP5SA$^{KI/+}$Col2a1creERT^{+ve}* biological replicates treated with 1 uM 4-hydroxytamoxifen (4-OHT) or ethanol vehicle (EtOH) at 24 hr after plating; E) 3 wild-type and 5 *Col2a1cre^{+ve}* biological replicates. Data were analysed by two-way ANOVA, with time elapsed and genotype as the independent variables, percentage apoptotic cells as the dependent variable. The effect of genotype on percentage apoptotic cells was not significant in any scenario. Scale bar = 150 µm (A).

F) Quantification of cell numbers following *in vitro* culture of primary chondrocytes from control (*Col2a1cre^{-ve}*), *Yap/Taz* double homozygous floxed animals, and nlsYap5SA-expressing animals. Related to Figure 1

Development • Supplementary information

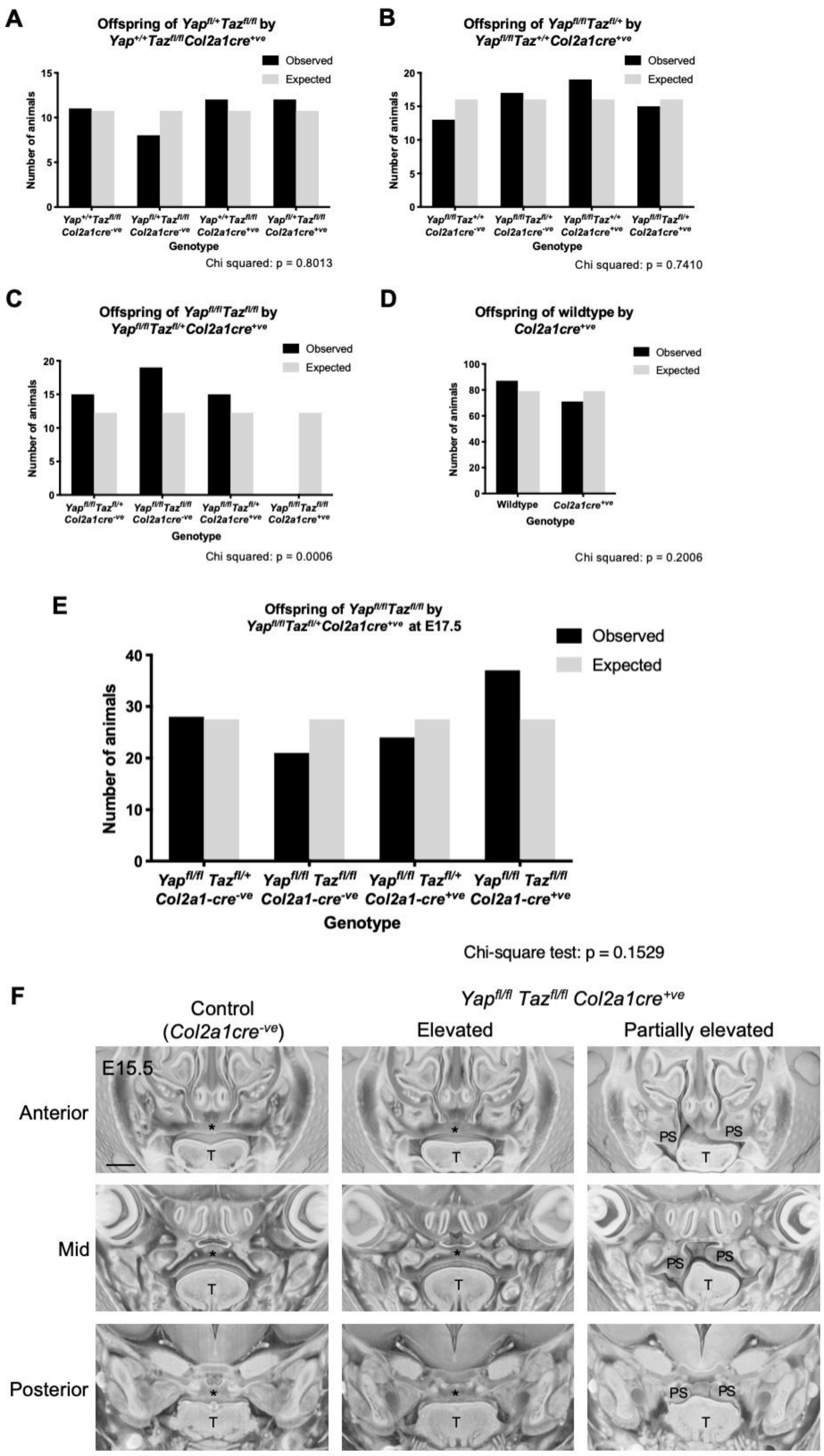

Development • Supplementary information

Figure S2. *Yap/Taz* double conditional mutants are not present at weaning owing to cleft palate.

A-E) Number of offspring of the indicated genotypes observed in crosses, versus the expected Mendelian ratio. Note the absence of the *Yap/Taz* double homozygous floxed animals in the presence of *Col2a1cre*. Genotypes of 2-3-week-old offspring of $Yap^{fl/+}Taz^{fl/fl}$ by $Yap^{+/+}Taz^{fl/fl}Col2a1cre^{+ve}$ (A), $Yap^{fl/fl}Taz^{fl/+}$ by $Yap^{fl/fl}Taz^{+/+}Col2a1cre^{+ve}$ (B), $Yap^{fl/fl}Taz^{fl/fl}$ by $Yap^{fl/fl}Taz^{fl/+}Col2a1cre^{+ve}$ (C) and wild type by $Col2a1cre^{+ve}$ (D). Note only $Yap^{fl/fl}Taz^{fl/fl}Col2a1cre^{+ve}$ animals (C) are absent at this timepoint, whereas these animals were present at expected numbers at E17.5 (E). Data were analysed by Chi squared tests. (F) Frontal cut-away of high resolution episcopic microscopy (HREM) 3-dimensional renderings of control ($Col2a1cre^{-ve}$) and $Yap^{fl/fl}Taz^{fl/fl}Col2a1cre^{+ve}$ foetuses at E15.5 along the anterior-posterior length of the palate. The palate (*) was closed in all 3 controls whereas the palates of 2 of 4 mutants were elevated and fused, while the palatal shelves (PS) of the remaining 2 mutants were partially elevated. T, tongue.
Scale bar = 0.5 mm.

Development • Supplementary information

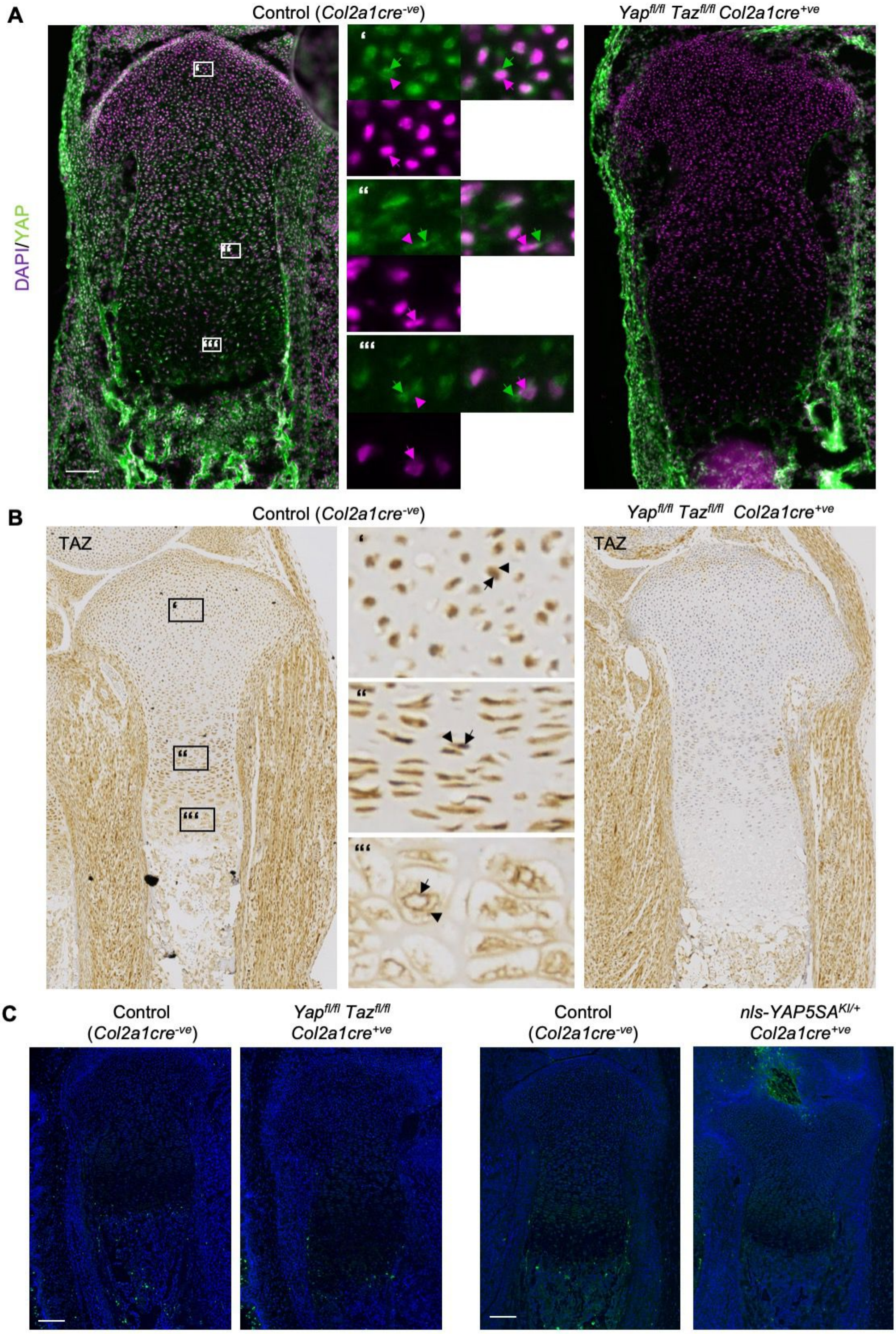
A
Control (Col2a1cre-ve)
Yapfl/fl Tazfl/fl Col2a1cre+ve
DAPI/YAP
B
Control (Col2a1cre-ve)
Yapfl/fl Tazfl/fl Col2a1cre+ve
TAZ
TAZ
C
Control (Col2a1cre-ve)
Yapfl/fl Tazfl/fl Col2a1cre+ve
Control (Col2a1cre-ve)
nls-YAP5SAKI/+ Col2a1cre+ve

Figure S3. Growth plate expression of YAP and TAZ in control and dKO growth plates and no change in apoptosis in dKO growth plates.

A) AP immunostaining (green) is present in the nucleus and cytoplasm of control tibial growth plates but not in the *Yap/Taz* double homozygous floxed animals in the presence of *Col2a1cre.* DAPI (purple) marks nuclei.

B) TAZ immunostaining (brown) is present in the nucleus and cytoplasm of control tibial growth plates but not in the *Yap/Taz* double homozygous floxed animals in the presence of *Col2a1cre.* Eosin (light blue) marks nuclei.

C) TUNEL staining of proximal growth plate of the tibia of E17.5 control (*Col2a1cre*$^{-ve}$) and *Yap*$^{fl/fl}$*Taz*$^{fl/fl}$*Col2a1cre*$^{+ve}$ pups and control (*Col2a1cre*$^{-ve}$) and *nls-YAP5SA*$^{KI/+}$*Col2a1cre*$^{+ve}$ pups. Images are representative of *n*=4 control (*Col2a1cre*$^{-ve}$), *n*=4 *Yap*$^{fl/fl}$*Taz*$^{fl/fl}$*Col2a1cre*$^{+ve}$ and *n*=3 *nls-YAP5SA*$^{KI/+}$*Col2a1cre*$^{+ve}$ E17.5 tibial growth plates.

Scale bar = 200 µm

Development • Supplementary information

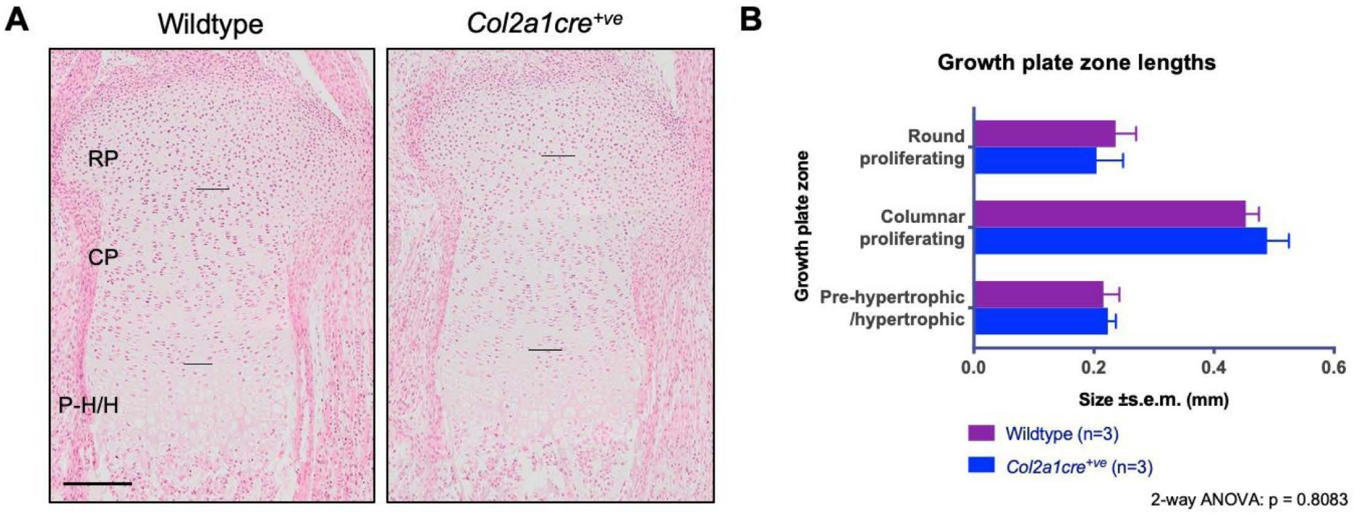

Figure S4. The *Col2a1cre* allele does not affect tibial growth plate zone size.

A) Tibial growth plates from both Wild-type and Col2a1cre-positive animals show no differences in growth plate size.

B) Quantification of zone lengths in A.

Development • Supplementary information

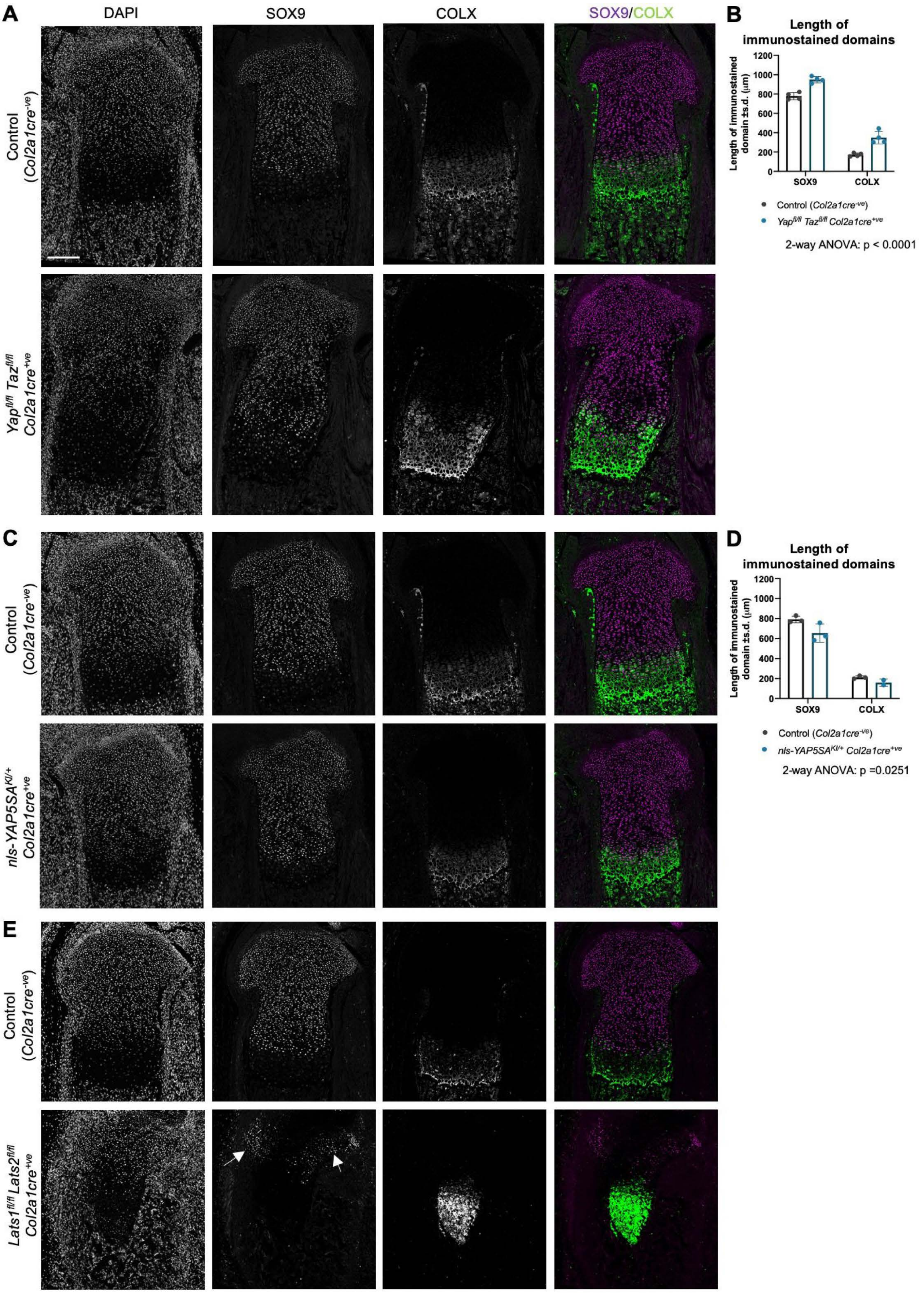
A
DAPI
SOX9
COLX
SOX9/COLX
Control (Col2a1cre-ve)
Yapfl/fl Tazfl/fl Col2a1cre+ve
B
Length of immunostained domains
Length of immunostained domain ±s.d. (μm)
1200
1000
800
600
400
200
0
SOX9
COLX
Control (Col2a1cre-ve)
Yapfl/fl Tazfl/fl Col2a1cre+ve
2-way ANOVA: p < 0.0001
C
Control (Col2a1cre-ve)
nls-YAP5SAKI/+ Col2a1cre+ve
D
Length of immunostained domains
Length of immunostained domain ±s.d. (μm)
1200
1000
800
600
400
200
0
SOX9
COLX
Control (Col2a1cre-ve)
nls-YAP5SAKI/+ Col2a1cre+ve
2-way ANOVA: p =0.0251
E
Control (Col2a1cre-ve)
Lats1fl/fl Lats2fl/fl Col2a1cre+ve

Figure S5. Chondrocyte marker expression in YAP/TAZ-modulated chondrocytes

A) Cartilage-specific double knockout of Yap/Taz does not affect expression of SOX9 or COLX in tibial growth plates.

B) Cartilage-specific double knockout of Yap/Taz increases the length of the immunostained domains in growth plates, consistent with reduced cell density.

C) Cartilage-specific expression of active nlsYAP^{5SA} does not affect expression of SOX9 or COLX in tibial growth plates.

D) Cartilage-specific expression of active nlsYAP^{5SA} decreases the length of the immunostained domains in growth plates, consistent with increased cell density.

E) Cartilage-specific double knockout of *Lats1/2* reduces overall size and reduces SOX9 expression levels, which may reflect a very strong activation of both YAP and TAZ.

Table S1: Primers for RTqPCR

Gene name	Primer sequences/Catalogue number	Source
*Yap**	QuantiTect: QT01061130	Qiagen
Taz	Fwd: 5’-GGGTTAGGGTGCTACAGTGT-3’<br>Rev: 5’-CTGACCGGAATTTTCACCTGT-3’	This study
Sox6	Fwd: 5’-GGAGATGCGACAGTTCTTCAC-3’<br>Rev: 5’-TCTGAGGTGATGGTGTGGTC-3’	This study
Sox9	Fwd: 5’-GACTCCCCACATTCCTCCTC-3’<br>Rev: 5’-CTGCTCAGTTCACCGATGTC-3’	This study
Ctgf	QuantiTect: QT00096131	Qiagen
Cyr61	QuantiTect: QT00245217	Qiagen
Acan	QuantiTect: QT00175364	Qiagen
Comp	Fwd: 5’-CCTGGGTGTCTTCTGCTTCT-3’<br>Rev: 5’-CCCTAGACTCTCTGCAGCC-3’	This study
Col2a1	Fwd: 5’-AAGTCACTGAACAACCAGATTGAGA-3’<br>Rev: 5’-AAGTGCGAGCAGGGTTCTTG-3’	Shea et al, 2019
Col10a1	Fwd: 5’-TGCAATCATGGAGCTCACAGA-3’<br>Rev: 5’-CAGAGGAGTAGAGGCCGTTTGA-2’	Shea et al, 2019
Mmp2	Fwd: 5’-GATGCTGCCTTTAACTGGAGT-3’<br>Rev: 5’-ACCGGGGTCCATTTTCTTCT-3’	This study
Mmp14	Fwd: 5’-GGGTCATTCATGGGCAGTGA-3’<br>Rev: 5’-CGCAGAGCTGACTTGGGATA-3’	This study
Mmp16	Fwd: 5’-GGTGGGAAGATGTTGGCAAA-3’<br>Rev: 5’-GGTGATGGGCTTGGGGTAA-3’	This study
Ctsk	Fwd: 5’-CAGAAGGGAAGCAAGCACTG-3’<br>Rev: 5’-ATTCCGAGCCAAGAGAGCAT-3’	This study
Hsp90ab1	Fwd: 5’-AGAATCCGACACCAAACTGC-3’<br>Rev: 5’-ACCTGGGAACCATTGCTAAG-3’	Voss et al., 2012

*Whilst this primer is listed as mouse-specific, we observed cross-reactivity with the human *YAP* sequence.

Vanyai et al Supplementary References

Shea, C.A., Rolfe, R.A., McNeill, H., and Murphy, P. (2020). Localization of YAP activity in developing skeletal rudiments is responsive to mechanical stimulation. Dev Dyn *249*, 523-542.

Voss, A.K., Vanyai, H.K., Collin, C., Dixon, M.P., McLennan, T.J., Sheikh, B.N., Scambler, P., and Thomas, T. (2012). MOZ regulates the Tbx1 locus, and Moz mutation partially phenocopies DiGeorge syndrome. Dev Cell *23*, 652-663.

Development • Supplementary information
