# Supplementary material for: Control of skeletal morphogenesis by the Hippo-YAP/TAZ pathway
Source: Development. 2020 Nov 12;147(21):dev187187. doi: 10.1242/dev.187187 (PMC7673359; doi:10.1242/dev.187187)
Supplement: Reviewer comments [file develop-147-187187_review_history.pdf]

Control of skeletal morphogenesis by the Hippo-YAP/TAZ pathway

Hannah K. Vanyai, Fabrice Prin, Oriane Guillermin, Bishara Marzook, Stefan Boeing, Alexander Howson, Rebecca E. Saunders, Thomas Snoeks, Michael Howell, Timothy J. Mohun and Barry Thompson
DOI: 10.1242/dev.187187

Editor: Sally Dunwoodie

Review timeline

Original submission:	6 December 2019
Editorial decision:	30 January 2020
First revision received:	11 June 2020
Editorial decision:	14 July 2020
Second revision received:	4 September 2020
Accepted:	7 September 2020

Original submission

First decision letter

MS ID#: DEVELOP/2019/187187

MS TITLE: Control of skeletal morphogenesis by the Hippo pathway

AUTHORS: Hannah Vanyai, Fabrice Prin, Stefan Boeing, Alexander Howson, Rebecca Saunders, Thomas Snoeks, Michael Howell, Timothy J Mohun, and Barry Thompson

I have now received all the referees' reports on the above manuscript, and have reached a decision. The referees' comments are appended below, or you can access them online: please go to BenchPress and click on the 'Manuscripts with Decisions' queue in the Author Area.

As you will see, the referees express considerable interest in your work, but have some significant criticisms and recommend a substantial revision of your manuscript before we can consider publication. If you are able to revise the manuscript along the lines suggested, which may involve further experiments, I will be happy to receive a revised version of the manuscript. Your revised paper will be re-reviewed by one or more of the original referees, and acceptance of your manuscript will depend on your addressing satisfactorily the reviewers' major concerns. Please also note that Development will normally permit only one round of major revision.

Please attend to all of the reviewers' comments and ensure that you clearly highlight all changes made in the revised manuscript. Please avoid using 'Tracked changes' in Word files as these are lost in PDF conversion. I should be grateful if you would also provide a point-by-point response detailing how you have dealt with the points raised by the reviewers in the 'Response to Reviewers' box. If you do not agree with any of their criticisms or suggestions please explain clearly why this is so.

© 2020. Published by The Company of Biologists under the terms of the Creative Commons Attribution License (https://creativecommons.org/licenses/by/4.0/).

Reviewer 1

Advance summary and potential significance to field

The manuscript reports the phenotypes of Yapf/fTazf/fCreCol2a1 embryos in which Yap and Taz activity has been inactivated within developing endochondral bones. The authors also report inactivation of Lats1&2 (which inhibit Yap and Taz) using the same Cre line and the effect of overexpression of Yap.

Novelty of manuscript:
Combined inactivation of both Yap and Taz during cartilage development.
Inactivation of Lats1&2 during cartilage development Cleft palate phenotype Widening of the cranial base

Comparison with other studies::
Deng et al. Yap knockout: increased skeletal length, increased mineralisation, decreased proliferation This study: Yap/Taz knockout: increased skeletal length, no change in mineralisation or proliferation Same stages analysed.

Deng et al. Post-natal stages, Yap overexpression: smaller growth plate, increased proliferation This study: E18.5, Yap overexpression: smaller growth plate, no change in proliferation

Goto et al. Mob1ab knockout: decreased Sox9, decreased mineralisation.
This study: Lats1/2 knockout: decreased Sox9, decreased mineralisation.; Yap overexpression or Yap/Taz knockout- no change in Sox9 expression.

In this study, the authors show the change in growth plate length is linked to alterations in cell density

In summary, there is some new data e.g. altered chondrocyte cell density and differences in cell proliferation and mineralisation data when compared to previous publications. The authors also report a cleft palate and cranial base phenotype, although these phenotypes have not been analysed in depth.

Apart from altered matrix production, from the current manuscript it is still hard to piece together the roles of Yap and Taz, including their potentially independent roles in the regulation of proliferation and differentiation. (this may explain some differences with previous publications). The authors can not assume Yap and Taz always act redundantly - this is often true but there is now clear evidence in several cell types, that they have differential and opposing functions - including in chondrocytes and osteoblasts. There are still questions. In the Lats1/2 knockout, Sox9 expression is decreased. This does not occur when Yap is overexpressed. Is this because Taz (and not Yap) inhibits Sox9 or the mouse model does not express Yap at sufficient levels?

Comments for the author

The manuscript reports the analysis of the roles of Yap and Taz during cartilage development. The data overlap with previous studies, add some new information and disagree with some previous reports (see below).
In several novel areas, the manuscript lacks depth. For example, the mechanism of the cleft palate phenotype is not concluded. The vertebrae appear to show a major defect but how their development is affected has not been characterised. The morphology (widening of the cranial base) is reported but the mechanism of this is not analysed.

One key issue that needs to be addressed throughout this manuscript is the control mouse model – this must be a Cre+ embryo or the authors must show that the Col2a1Cre line does not have any non-specific effects due to the Cre recombinase. Although I appreciate some comparison of Yapf/fCre+ versus Yapf/f/Tazf/fCre data is presented e.g. Fig. 4, this is essential for each of studies e.g. proliferation, histology.

© 2020. Published by The Company of Biologists under the terms of the Creative Commons Attribution License (https://creativecommons.org/licenses/by/4.0/).

Specific Comments:
Major:
1. Can the authors confirm that the Cre recombinase does not have an effect. For example in Fig1. A Cre+ control is needed e.g. Yapf/fTazf/+Cre to eliminate any non-specific effects of the Cre. Or show that the Cre has no effect in the supplementary material 2. Given that there are differences in cell adherence, measuring the degree of confluence cannot be used to analyse differences in proliferation in vitro (Figs.1B,D)
3. The cartilage staining in the skeletal images in Figure 2 is a little unclear. The vertebrae seem to be more severely affected judging by the decreased ossification – do the authors have clearer images. A defect in vertebrae development may also account for the altered rib and sternum morphology which may be bent because the thoracic vertebrae are closer together.
4. The conclusion that the cleft palate phenotype is due to widening of the cranial base is an assumption.
Cleft palate can arise due to a failure of Meckels cartilage to develop appropriately. A defect in Meckels cartilage decreases growth of the lower jaw and the secondarily lowering of the tongue which allows elevation of the palatal shelves. The statement (line 219) that the tongue is tightly wedged in the palatal shelve indicates that the tongue has not lowered. The statement that the cranial base phenotype is deformed in all mutants irrespective of the cleft palate phenotype (line 224) is also inconsistent with the cranial base phenotype being causative of the cleft palate phenotype (also see Fig. 3R).
The authors need to examine development of Meckel's cartilage and carry out a series of histological studies showing the specific stage of palatal shelf development that is affected i.e. growth and elevation. Where is the tongue in relation to the palatal shelves? This study will show (a) if there is delay in palatal shelf elevation due to obstruction by the tongue or (b) the palatal shelves and tongue develop normally but the palatal shelves fail to meet – potentially due to a widening of the upper jaw/cranial base.
5. The authors also need to describe briefly how the palatal shelves develop (to help readers without any prior knowledge) and discuss the cleft palate phenotype, particularly as Yap mutations are associated with cleft palate in humans and that this is one of the reasons for undertaking the analyses.
6. The graphs need to be represented to show the individual datapoints from each biological replicate.
7. Lines 199& 352.I disagree that the study argues that Yap/Taz has a primary role in morphogenesis. Yap and Taz may have distinct functions (and potentially opposing) which could potentially be masked in this study.
8. There needs to be quantitative analysis i.e. measurement of length of Sox9 and type X collagen expressing domains in Figure 7.
9. Can the authors show where Taz is expressed. Ideally, a comparison of the Yap and Taz expression domains (and activity i.e. nuclear localisation) would be helpful, although I appreciate the Yap expression domain has already been published.
10. I would have liked to have seen more molecular characterisation of the growth plates. For the proliferation analysis, it would have been helpful to see proliferation in the yap knockout versus the Taz knockout mice in case Yap and Taz have distinct roles.

Minor:
11. The difference in flattening in Fig 1A is clear at 93 hours. The authors propose that this is due to differences in integrin-matrix binding interactions. Do the authors have any qPCR data to support this proposal e.g. adherens junctions components that are targets of Yap/Taz. Alternatively, could this potentially relate to altered differentiation?
12. The differences in vitro versus in vivo are probably not surprising. In the former, the cells are relatively sparse with limited cell-to-cell contact. In this situation, Yap/Taz activity would be increased. The differences between this study and study with cell lines may also be related to cell density effects.
13. Fig. 1B. The green and pink lines show the same genotype (Yapf/f/Tazf/+Col2a1Cre) – is this correct?
14. Line 606 (Figure legend 2). The effect of genotype on length was insignificant.
15. Line 191. The microCT measures the length of mineralisation (which may or may not reflect the length of the skeletal element). Therefore, change "length of bone" to "length of mineralisation".
16. For clarity label the growth plate zones in the figures.

© 2020. Published by The Company of Biologists under the terms of the Creative Commons Attribution License (https://creativecommons.org/licenses/by/4.0/).

17. Figure legend 4. Lines 646-648. Change cell number to cell density. As written, this is incorrect.
18. Figures 4E and 4H show the same information from different biological replicates. This data should be combined.
19. Fig. 4L. Line 673. Clarify if the change in matrix levels is significantly changed in all regions of the growth plate or within some zones of the growth plate.
20. Line 311. Recent fate mapping studies have indicated that hypertrophic chondrocytes give rise to osteoblasts within the bone e.g. see Kathy Cheahs publication. Therefore, they do not “clear” as classically thought.

Reviewer 2

Advance summary and potential significance to field

The authors present new genetic models of yap signaling modulation in the developing cartilage using a Col2-cre allele for the purposes of clarifying the role of this pathway in chondrocyte proliferation, maturation and bone morphogenesis. The generation of double knockout mice for both Yap and Taz provides insightful information regarding the in vitro and in vivo roles of these factors, building on what has been shown previously using single knockout. The approach to study chondrocyte biology both in vitro and in vivo is commended, as these cells remarkably can change their phenotype and function once removed from their native tissue matrix. This manuscript is of very high quality and will be of interest to the development and skeletal biology communities.

Comments for the author

Major comment
The evaluation (and conclusions based on this parameter) of percentage of extracellular matrix is inadequate.
Alcian blue staining is a qualitative indicator of the presence of sulfated proteoglycans and should not be used histologically to quantify ECM content. Further, the persistence of ECM in the hypertrophic zone indicates that there is a delay in terminal differentiation of these cells, as the matrix is not sufficiently remodeled into bone, perhaps suggesting that the cells themselves are not providing the necessary signals to the surrounding tissue to promote remodeling efficiently. Typically what is done in the skeletal biology field to indicate defects in ECM production are immunostaining for traditional ECM markers of different zones which is highlighted in this paper in Figure 7. An expansion of ColX staining would support the notion that the hypertrophic chondrocyte zone is expanded, persisting, but not that ECM production is downregulated (or upregulated as inferred in Figure 5 and associated text). If the authors believe that misregulation or increased/decreased ECM production (per chondrocyte) is causing these phenotypes, they should quantify this by gene expression, biochemical assays (DMMB, hydroxyproline, solubilization of alcian blue, all normalized to DNA content), elisa, or quantify ECM production (gene expression/biochemistry) in vitro using pellet cultures of rib chondrocytes. In my opinion, should the authors choose to make less conclusive statements regarding the ECM quantity, the remaining data and conclusions are excellent and sufficient for the scope of this manuscript.

Minor comments

In the long bones histologically at E17.5, an expanded hypertrophic zone was detected and quantified. The authors detect minor but significant changes in cell density in two different stained samples/sections. These values may change depending on what section and how many were chosen for counting, thus it would be beneficial to have this information in the methods section.

In Figure 4A it would be helpful to add bars alongside each image that visually denote the 3 different zones of cartilage the authors quantify.

© 2020. Published by The Company of Biologists under the terms of the Creative Commons Attribution License (https://creativecommons.org/licenses/by/4.0/).

Reviewer 3

Advance summary and potential significance to field

In this manuscript, the authors study the Hippo pathway in the developing skeleton. Using Col2-Cre as a deleter, the authors execute an informative collection of gain- and loss-of-function experiments with aim to understand the function of this pathway in the development of the skeleton. The authors describe a set of deformation phenotypes and rule out the effect of chondrocyte proliferation or differentiation in these phenotypes. Thus, they conclude that the Hippo pathway functions in controlling cartilage morphogenesis.

Comments for the author

While the research question is important and the findings are novel and interesting, I think that the substantiation of the conclusion is inadequate. First, the authors use the Col2-Cre line which, if I am not mistaken, starts to have an observable effect at E13.5. Therefore, the authors should examine their mutants around this time, at E13.5-E14.5. Second, the authors describe a cleft palate phenotype (if the shelves are elevated or not), which should also be studied at that time point. Lastly, osteoblasts of the cranial dermal bone do express Col2 (Abzhanov A1, Rodda SJ, McMahon AP, Tabin CJ. Development. 2007 Sep;134(17):3133-44. Epub 2007 Aug 1). This can lead to Cre activity in this bone, which could affect the interpretation of the results. Notably, Col2-Cre has a tamoxifen-induced version (Col2-CreER), which provides temporal control and thereby the ability to dissect the effect of the Hippo pathway at different stages.
To conclude, while it would not be reasonable to require the authors to provide a mechanism of action for the observed phenotypes, to merit publication in Development a more complete and thorough developmental analysis must be provided, and not just the prenatal end result.

First revision

Author response to reviewers' comments

Response to the Reviewers' comments

We sincerely thank the reviewers for their positive comments on the manuscript and their constructive suggestions, which have improved the manuscript. The revised version contains substantial new data and figures to address the comments of both reviewers. We now feel that our manuscript is suitable for publication in Development. We provide a point-by-point response to their comments below.

Reviewer 1
Major comment 1:
Can the authors confirm that the Cre recombinase does not have an effect. For example in Fig1. A Cre+ control is needed e.g. Yapf/fTazf/+Cre to eliminate any non-specific effects of the Cre. Or show that the Cre has no effect in the supplementary material.

We understand the concern of the reviewer's comment, which they also refer to in the general comments, that the control mouse model "must be a Cre+ embryo or the authors must show that the Col2a1Cre line does not have any non-specific effects due to the Cre recombinase". We agree with the reviewer that this is especially true of the in vitro work and we have therefore performed the primary chondrocyte culture assay comparing wildtype and Col2a1cre+ve genotypes (Fig. S1). This demonstrates that the Col2a1cre transgene alone does not affect the in vitro growth of chondrocytes.
Regarding the in vivo work, no phenotype has been reported in animals carrying a single Col2a1cre transgene, though subtle cranial deformities have been reported in animals carrying two copies of the transgene (https://journals.plos.org/plosone/article?id=10.1371/journal.pone.0066206#s2). Here we see no phenotype at the gross morphological level nor the histological level of the tibial

© 2020. Published by The Company of Biologists under the terms of the Creative Commons Attribution License (https://creativecommons.org/licenses/by/4.0/).

growth plate in any Col2a1cre-carrying mutant with any Yap;Taz floxed allelic combination examined aside from the double homozygous mutant (Fig 4a) and we have also now included wildtype and Col2a1cre+ve animals, which show no difference, in a new supplementary figure (Fig. S4). Nor is there any difference in viability except in the Yapfl/flTazfl/flCol2a1cre+ve animals in the post-natal period and we have included a new supplementary figure to reflect this which includes histology of Col2a1cre+ve animals (Fig. S2).
We are therefore confident that the in vivo phenotypes we report here are a result of loss of all four Yap;Taz alleles rather than an effect of the Col2a1cre transgene. With this in mind, we believe that using Col2a1cre-ve littermates is of more value to control for litter-to-litter variability than generating Col2a1cre+ve animals for use as controls, especially in our mouse strain that is on a mixed background in which we notice greater litter-to-litter developmental variability than we have experienced on inbred strains. Furthermore, for our molecular analysis by RTqPCR (Fig. 7A), we have included the Yapfl/flTazfl/+Col2a1cre+ve genotype in addition to Col2a1cre-ve samples for additional reassurance that the observed molecular changes are likely to be causative of the phenotypes observed in Yapfl/flTazfl/flCol2a1cre+ve pups and are not present in phenotypically normal, Col2a1cre-carrying littermates (Yapfl/flTazfl/+Col2a1cre+ve pups).

Major comment 2:
Given that there are differences in cell adherence, measuring the degree of confluence cannot be used to analyse differences in proliferation in vitro (Figs.1B,D).

If we understand the comment correctly, the concern is that a difference in cell adherence may affect the degree of confluence because of cells detaching during media changes and movement of the plate. However, in this experimental set up, both these factors are minimised as a) the media is not changed through the duration of the measurements and b) the plate is not moved once placed into the IncuCyte unit within the incubator and it is the camera which moves around to photograph the plate. The only movement of the plate occurs when the drawer of the IncuCyte unit is (infrequently) opened by another user to access other plates. A simple count of cell numbers confirms that most of the difference in confluence can be attributed to the number of cells rather than changes in cellular surface area.

Major comment 3:
The cartilage staining in the skeletal images in Figure 2 is a little unclear. The vertebrae seem to be more severely affected judging by the decreased ossification – do the authors have clearer images. A defect in vertebrae development may also account for the altered rib and sternum morphology which may be bent because the thoracic vertebrae are closer together.

We agree that the vertebrae appear to be more severely affected in the Yapfl/flTazfl/flCol2a1cre+ve animals than other bones and they certainly are extremely malformed in mutant models of increased YAP or YAP/TAZ activity. As such, we believe that examination of the Hippo pathway in vertebral development is worthy of its own project and have not attempt to explore it further here. We have chosen instead to focus on the cleft palate phenotype and we provide new data to show a defect in development of Meckel's cartilage is likely the primary mechanism, with possible contribution from a widened cranial base.

Major comment 4:
The conclusion that the cleft palate phenotype is due to widening of the cranial base is an assumption. Cleft palate can arise due to a failure of Meckels cartilage to develop appropriately. A defect in Meckels cartilage decreases growth of the lower jaw and the secondarily lowering of the tongue which allows elevation of the palatal shelves. The statement (line 219) that the tongue is tightly wedged in the palatal shelve indicates that the tongue has not lowered. The statement that the cranial base phenotype is deformed in all
mutants irrespective of the cleft palate phenotype (line 224) is also inconsistent with the cranial base phenotype being causative of the cleft palate phenotype (also see Fig. 3R).
The authors need to examine development of Meckel's cartilage and carry out a series of histological studies showing the specific stage of palatal shelf development that is affected i.e. growth and elevation. Where is the tongue in relation to the palatal shelves? This study will show (a) if there is delay in palatal shelf elevation due to obstruction by the tongue or (b) the palatal shelves and tongue develop normally but the palatal shelves fail to meet – potentially due to a widening of the upper jaw/cranial base.

© 2020. Published by The Company of Biologists under the terms of the Creative Commons Attribution License (https://creativecommons.org/licenses/by/4.0/).

In our initial submission, we had attributed the cleft palate to widening of the cranial base as we had not observed a defect in the size or shape of the mandibles in skeletal preparations of E17.5 mutants with unelevated cleft palates and we now included these data as Fig. 3P,Q. Though we acknowledge it is not a widely discussed mechanism for cleft palate, Ivkovic et al. attribute the cleft palate observed in Ctgf knockout mutants (which our mutants phenocopy, see new text) as being “most likely as a secondary consequence of defects in the formation of endochondral elements at the base of the skull and in nasal cartilages” (Ivkovic et al 2003, Development). Rather than being due to the palatal shelves being unable to meet, this may impede elevation by widening the oral-nasal cavity, in turn increasing the space which the tongue can occupy and therefore the palatal shelves may capture more of the tongue as they attempt to rise. We had hoped to provide E13.5 HREM analysis of the position of the tongue in the oral-nasal cavity relative to the palatal shelves prior to elevation, unfortunately this was unable to be completed due to current circumstances. In some human patients with cleft lip and palate, anomalies of the cranial base are believed to be causative for the clefting (https://doi.org/10.1080/00016350510019847).
However, we have now also used high resolution episcopic microscopy (HREM) to examine palate development at E14.5 and E15.5. At E14.5 (Fig. 3R), it is apparent that there is a delay in elevation in the mutants and the tongue is high in the oral-nasal cavity. There is also a substantial deformation of Meckel’s cartilage at this stage (extracted images in Fig. 3S), including being shorter in the anterior-posterior axis. It is therefore likely that cleft palate observed in our mutants arises from defects in the development of Meckel’s cartilage during the stages of palate development crucial for palatal shelf elevation. Though we do not dismiss changes to the cranial base as a possible contributing mechanism to the cleft palate observed, we have chosen to focus on Meckel’s cartilage deformation as the more commonly recognised cause of cleft palate.

Major comment 5:
The authors also need to describe briefly how the palatal shelves develop (to help readers without any prior knowledge) and discuss the cleft palate phenotype, particularly as Yap mutations are associated with cleft palate in humans and that this is one of the reasons for undertaking the analyses.
As suggested, we have included discussion of palate development and the cleft palate phenotype.

Major comment 6:
The graphs need to be represented to show the individual datapoints from each biological replicate.
Thank you, the graphs throughout have been remade to display individual biological replicate data points.

Major comment 7:
Lines 199& 352.I disagree that the study argues that Yap/Taz has a primary role in morphogenesis. Yap and Taz may have distinct functions (and potentially opposing) which could potentially be masked in this study.
We think the possibility that YAP and TAZ may have distinct functions is really fascinating and as the reviewer notes in their initial summaries “In the Lats1/2 knockout, Sox9 expression is decreased. This does not occur when Yap is overexpressed. Is this because Taz (and not Yap) inhibits Sox9 or the mouse model does not express Yap at sufficient levels?”. In our revised manuscript, we do not exclude this possibility. Certainly, a TAZ equivalent of the constitutively nuclear nls-YAP5SA would be the obvious starting point, but such a transgenic mouse line is not currently available. Nevertheless, in the conditional knockout mutants (Yapfl/flTazfl/flCol2a1cre+ve) a number of observations argues for predominantly redundant roles for YAP and TAZ in chondrogenesis in vivo. First and foremost, we only observed phenotypes in Yapfl/flTazfl/flCol2a1cre+ve mutant animals. In other words, the presence of one allele of either Yap (Yapfl/+Tazfl/flCol2a1cre+ve) or Taz (Yapfl/flTazfl/+Col2a1cre+ve) was sufficient to avoid a) neonatal lethality (new Fig. S2); b) gross morphology defects (mentioned in text); c) the elongated growth plate (Fig. 4A,B); d) decreased cell density (data not shown) and e) molecular changes, at least for the mutant we examined (Yapfl/flTazfl/+Col2a1cre+ve; Fig. 7A-E). Whilst we cannot rule out distinct functions leading to subtle effects in single mutant animals, we chose to focus on the most striking phenotypes, which were observed only in the Yapfl/flTazfl/flCol2a1cre+ve animals.

© 2020. Published by The Company of Biologists under the terms of the Creative Commons Attribution License (https://creativecommons.org/licenses/by/4.0/).

Major comment 8:
There needs to be quantitative analysis i.e. measurement of length of Sox9 and type X collagen expressing domains in Figure 7.
We now include graphs detailing the measurements of SOX9 and COLX expression domains in what is now Fig. S5.

Major comment 9:
Can the authors show where Taz is expressed. Ideally, a comparison of the Yap and Taz expression domains (and activity i.e. nuclear localisation) would be helpful, although I appreciate the Yap expression domain has already been published.
We now provide immunostaining for YAP and TAZ in the tibial growth plate in Fig. S3.

Major comment 10:
I would have liked to have seen more molecular characterisation of the growth plates. For the proliferation analysis, it would have been helpful to see proliferation in the yap knockout versus the Taz knockout mice in case Yap and Taz have distinct roles.
We have now included molecular characterisation of microdissected tibial growth plates by RTqPCR for Yapfl/flTazfl/flCol2a1cre+ve and Col2a1cre–ve samples, as well as Yapfl/flTazfl/+Col2a1cre+ve littermate controls, and nls-YAP5SAKI/+Col2a1cre+ve and Col2a1cre–ve controls, in a new Fig. 7. This has enabled us to clarify further that there is no effect on Sox9 nor Sox6 expression levels through modulation of YAP/TAZ in these mutants and has illustrated that known YAP/TAZ targets Ctgf and Cyr61 are significantly affected by loss of Yap/Taz (both) or overactivation of YAP (Ctgf only). We further clarified this loss of expression by in situ hybridisation in Yapfl/flTazfl/flCol2a1cre+ve tibias.
Regarding the proliferation analysis on single Yap and Taz knockout samples, we were unable to perform this experiment prior to the coronavirus lockdown. Nevertheless, we note that neither single knockout has a strong phenotype on its own, consistent with a largely redundant function during development.

Minor comment 11:
The difference in flattening in Fig 1A is clear at 93 hours. The authors propose that this is due to differences in integrin-matrix binding interactions. Do the authors have any qPCR data to support this proposal e.g. adherens junctions components that are targets of Yap/Taz. Alternatively, could this potentially relate to altered differentiation?
We agree with the reviewer that the difference in flattening may be due to altered differentiation and, as we do not have RTqPCR data to distinguish between the two alternative explanations, we have included altered differentiation as a possible explanation in the text. Nevertheless, published work indicates that YAP can transcriptionally mediate 'activation of genes encoding integrins and FA [focal adhesion] docking proteins' in cell culture (Nardone G et al. 2017 Nature Communications).

Minor comment 12:
The differences in vitro versus in vivo are probably not surprising. In the former, the cells are relatively sparse with limited cell-to-cell contact. In this situation, Yap/Taz activity would be increased. The differences between this study and study with cell lines may also be related to cell density effects.
We thank the reviewer for the thoughtful comments and agree that the disparity between reported roles of YAP/TAZ in chondrocytes in culture are likely to be influenced by factors such as cell density effects. We think that this is an important argument for caution in using in vitro models of YAP/TAZ activity in chondrocytes as the physiological relevance of conclusions drawn from such studies is questionable.

Minor comment 13:
Fig. 1B. The green and pink lines show the same genotype (Yapf/f/Tazf/+Col2a1Cre) – is this correct?
Yes, the reviewer has correctly noted that the green and pink lines show the same genotype, Yapfl/flTazfl/+Col2a1cre+ve. The data represent one experiment of four conducted independently with primary chondrocytes isolated from individual pups i.e. biological replicates. Three to four biological replicates were included per experiment and consisted of varying combinations of controls, homozygous mutants and Yapfl/flTazfl/+Col2a1cre+ve animals. This particular experiment

© 2020. Published by The Company of Biologists under the terms of the Creative Commons Attribution License (https://creativecommons.org/licenses/by/4.0/).

contained one control (Col2a1cre-ve), one homozygous mutant and two Yapfl/flTazfl/+Col2a1cre+ve littermates.

Minor comment 14:
Line 606 (Figure legend 2). The effect of genotype on length was insignificant.
Thank you for the reviewer's attention to detail. We have now corrected this typo.

Minor comment 15:
Line 191. The microCT measures the length of mineralisation (which may or may not reflect the length of the skeletal element). Therefore, change "length of bone" to "length of mineralisation".
We have clarified this statement as suggested.

Minor comment 16:
For clarity label the growth plate zones in the figures.
We have now denoted the borders of the cartilage zones in Fig. 4A,K, S4A, 5K and we appreciate this suggestion for the clarity it brings to the data point.

Minor comment 17:
Figure legend 4. Lines 646-648. Change cell number to cell density. As written, this is incorrect.
Thank you, we have corrected this statement.

Minor comment 18:
Figures 4E and 4H show the same information from different biological replicates. This data should be combined.
The data obtained for Fig. 4E and 4H show the same information from different biological replicates but obtained by two distinct methods and therefore represent two distinct datasets. Fig. 4E was obtained by manually counting IHC-counterstained nuclei. Fig. 4H was obtained through automated counting of DAPI-stained fluorescent nuclei. We have clarified this in the methods.

Minor comment 19:
Fig. 4L. Line 673. Clarify if the change in matrix levels is significantly changed in all regions of the growth plate or within some zones of the growth plate.
Unfortunately, the test does not have sufficient power to distinguish, by post-testing the 2-way ANOVA, whether the change in matrix levels is specific to certain zones and we recognise that this is a limitation of the experiment. Owing to the lockdown, we could repeat the experiment to answer this question.

Minor comment 20:
Line 311. Recent fate mapping studies have indicated that hypertrophic chondrocytes give rise to osteoblasts within the bone e.g. see Kathy Cheahs publication. Therefore, they do not "clear" as classically thought.
Thank you for the suggestions, we have clarified the statement and included references including that of Kathryn Cheah.

Reviewer 2:
Major comment:
The evaluation (and conclusions based on this parameter) of percentage of extracellular matrix is inadequate…If the authors believe that misregulation or
increased/decreased ECM production (per chondrocyte) is causing these phenotypes, they should quantify this by gene expression, biochemical assays (DMMB, hydroxyproline, solubilization of alcian blue, all normalized to DNA content), elisa, or quantify ECM production (gene expression/biochemistry) in vitro using pellet cultures of rib chondrocytes.
We thank the reviewer for their detailed suggestions and for stating that, with less conclusive comments on the point of ECM quantity, "the remaining data and conclusions are excellent and sufficient for the scope of this manuscript". We now provide new data in Fig. 7 to directly address the question of ECM production, specifically in the in vivo setting to avoid any possible non-physiological conclusions of an in vitro assay, given the discrepancy we see between the two systems. In microdissected tibial growth plate sections, RTqPCR analysis showed that there is no change in the expression of ECM component genes Acan, Comp, Col2a1 and Col10a1 with the loss of Yap and Taz or the overactivity of nls-YAP5SA (excepting Col10a1 expression which decreases),

© 2020. Published by The Company of Biologists under the terms of the Creative Commons Attribution License (https://creativecommons.org/licenses/by/4.0/).

showing that ECM protein production (per chondrocyte) is not negatively regulated by YAP/TAZ. Instead, we show new evidence that YAP/TAZ modulation results in substantial gene expression changes to known YAP/TAZ direct target genes Ctgf and Cyr61, which are known to regulate ECM remodelling in various tissues through modulation of ECM proteases such as CTGF regulation of Mmp2 in the heart (Mo and Lau, Circ Res, 2006) and Mmp9 in cartilage, (Ivkovic et al., Development, 2003). We therefore conclude that the increases in ECM area in Yapfl/flTazfl/flCol2a1cre+ve tibial growth plates are due to a decrease in protease activity and we demonstrate that Mmp2 and Ctsk, both encoding ECM proteases, are decreased in these samples. Conversely and correspondingly, Ctgf and Mmp16 are upregulated in the smaller tibial growth plates of YAP5SAKI/+Col2a1cre+ve pups. Therefore, we conclude that YAP/TAZ regulate cartilage remodelling by ECM turnover, via regulating Ctgf and Cyr61 and downstream proteases.

Minor comment 1:
In the long bones histologically at E17.5, an expanded hypertrophic zone was detected and quantified. The authors detect minor but significant changes in cell density in two different stained samples/sections. These values may change depending on what section and how many were chosen for counting, thus it would be beneficial to have this information in the methods section.
One tibial growth plate section per biological replicate was examined and we have now clarified this information in the methods section. Reassuringly, the fact we see significant changes in cell density by two approaches in two sets of biological replicates processed at very different times (approximately one year apart) speaks to the fact that the change in cell density is robust despite being relatively subtle.

Minor comment 2:
In Figure 4A it would be helpful to add bars alongside each image that visually denote the 3 different zones of cartilage the authors quantify.
We have now denoted the borders of the cartilage zones in Fig. 4A,K, S4A, 5K and we appreciate this suggestion for the clarity it brings to the data point.

Reviewer 3:
While the research question is important and the findings are novel and interesting, I think that the substantiation of the conclusion is inadequate. First, the authors use the Col2-Cre line which, if I am not mistaken, starts to have an observable effect at E13.5. Therefore, the authors should examine their mutants around this time, at E13.5-E14.5.
We appreciate that the reviewer believes our findings are novel and interesting and we hope that we have included additional data and explanations to convince them that the substantiations of our conclusions are adequate.
The reviewer is correct that Col2a1-cre is expressed earlier than examined here, from around E11.5 in the cartilaginous anlagen of the vertebrae and long bones (Ovchinnikov et al., Genesis, 2000). However, the rationale for focussing on the E17.5 timepoint is twofold. Firstly, examining animals at E17.5 enables us to see the gross morphological consequence, or not, of earlier deletion and therefore in what part of the skeleton YAP/TAZ may have a role. Secondly, the E17.5 timepoint also provides us with an in vivo model of chondrogenesis in the form of the tibial growth plate, which both enables us to examine all stages of chondrogenesis simultaneously (round proliferating, columnar proliferating, prehypertrophic, hypertrophic and terminally differentiated) and also avoid using in vitro differentiation models, which we are reluctant to use due to the extreme disparity in phenotype between in vitro and in vivo results we detail here. The tibial growth plate model provides a pseudo-temporal snapshot of chondrocyte development and as such, stage-specific defects in chondrogenesis can be inferred from the variation in size of growth plate zones, gene expression domains or regions of proliferation or apoptosis. We have added a description in the text of this explanation for our choice of the tibial growth plate as a model of chondrocyte differentiation.

Second, the authors describe a cleft palate phenotype (if the shelves are elevated or not), which should also be studied at that time point.
We thank the author for the important suggestion and we have now examined E14.5 and E15.5 foetuses by HREM analysis and determined that the cleft palate phenotype is caused by a delay in or impediment of palatal shelf elevation, likely due to morphological defects in Meckel’s cartilage at the stage crucial for palate elevation (E14.5), perhaps with some contribution from the defects

© 2020. Published by The Company of Biologists under the terms of the Creative Commons Attribution License (https://creativecommons.org/licenses/by/4.0/).

observed in the cranial base. This forms two new panels, Fig. 3R,S. We were unable to complete the E13.5 timepoint given current circumstances.

Lastly, osteoblasts of the cranial dermal bone do express Col2 (Abzhanov A1, Rodda SJ, McMahon AP, Tabin CJ. Development. 2007 Sep;134(17):3133-44. Epub 2007 Aug 1). This can lead to Cre activity in this bone, which could affect the interpretation of the results.
Thank you to the reviewer for alerting us to this publication. Whilst we do not rule out a role for Yap/Taz in Col2-expressing cranial dermal bone, we have provided clear evidence of defects in the cartilage-derived cranial base (original submission) as well as Meckel's cartilage (new data), both of which are able to contribute to the cleft palate phenotype and the delay in palatal shelf elevation evident in the E14.5 HREM data is consistent with the known mechanism of the tongue impacting the palatal shelves' capacity to elevate. Furthermore, the cranial dermal bone-derived mandibles are not malformed at E17.5 (new data in Fig. 3), suggesting that the lower jaw defects are primary due to defects with Meckel's cartilage and not mandible bone development.

Notably, Col2-Cre has a tamoxifen-induced version (Col2-CreER), which provides temporal control and thereby the ability to dissect the effect of the Hippo pathway at different stages.
To conclude, while it would not be reasonable to require the authors to provide a mechanism of action for the observed phenotypes, to merit publication in Development a more complete and thorough developmental analysis must be provided, and not just the prenatal end result.
We agree that the use of the Col2a1-creERT allele will be an important tool for further studies to dissect the stage-specific requirements of the Hippo pathway, however we believe this investigation goes beyond the scope of the study here.
We believe we have now provided evidence of a clear developmental mechanism for the observed cleft palate phenotype in the E14.5 HREM data, have demonstrated a cellular biological mechanism in the variation in area of ECM in our tibial growth plate model of chondrogenesis and furthermore have provided a molecular mechanism of dysregulation of chondrogenesis in mutants with YAP/TAZ modulation through the RTqPCR analysis of laser microdissected tibial growth plates. We hope the reviewer will now feel that we have provided sufficient data to substantiate our "novel and interesting" findings.

Second decision letter

MS ID#: DEVELOP/2019/187187

MS TITLE: Control of skeletal morphogenesis by the Hippo pathway

AUTHORS: Hannah Vanyai, Fabrice Prin, Oriane Guillermin, Bishara Marzook, Stefan Boeing, Alexander Howson, Rebecca Saunders, Thomas Snoeks, Michael Howell, Timothy J Mohun, and Barry Thompson

I have now received all the referees' reports on the above manuscript, and have reached a decision. The referees' comments are appended below, or you can access them online: please go to BenchPress and click on the 'Manuscripts with Decisions' queue in the Author Area.

As you will see, the referees appreciated the changes that you made to the manuscript. However, there are still some underlying issues that are of concern.

Reviewer 1 has raised 11 issues. Please address all issues, #9 is optional.
Reviewer 2 remains concerned about primary direct effects verses secondary consequences of YAP/TAZ deletion. I understand that it is not currently possible to examine earlier developmental stages. I ask that you alter the text so it is clear that phenotyping at E17.5 does not discern the direct early effects of YAP/TAZ deletion and that to address this earlierstages of development would need to be examined.

If you are able to revise the manuscript along the lines suggested, I will be happy to receive a revised version of the manuscript. Your revised paper will be re-reviewed by one or more of the

© 2020. Published by The Company of Biologists under the terms of the Creative Commons Attribution License (https://creativecommons.org/licenses/by/4.0/).

original referees, and acceptance of your manuscript will depend on your addressing satisfactorily the reviewers' major concerns.

We are aware that you may currently be unable to access the lab to undertake experimental revisions. If it would be helpful, we encourage you to contact us to discuss your revision in greater detail. Please send us a point-by-point response indicating where you are able to address concerns raised (either experimentally or by changes to the text) and where you will not be able to do so within the normal timeframe of a revision. We will then provide further guidance. Please also note that we are happy to extend revision timeframes as necessary.

Please attend to all of the reviewers' comments and ensure that you clearly highlight all changes made in the revised manuscript. Please avoid using 'Tracked changes' in Word files as these are lost in PDF conversion. I should be grateful if you would also provide a point-by-point response detailing how you have dealt with the points raised by the reviewers in the 'Response to Reviewers' box. If you do not agree with any of their criticisms or suggestions please explain clearly why this is so.

Reviewer 1

Advance summary and potential significance to field

The manuscript describes the role of Yap and Taz during chondrocyte development using loss of function
(double mutant) and gain of function strategies (Yap constitutively active and double Lats1/2 knockout). The authors show that Yap/Taz primarily regulate matrix levels, identifying a few potential regulators/direct targets, and that there is a cleft palate phenotype in the Yap/Taz mutants which is secondary to a defect in Meckel's cartilage.

A change of title may be appropriate. Yap/Taz are not just regulated by the Hippo pathway, although I appreciate that the Lats1/2 mutant shows the same phenotype as constitutively active Yap so I understand why the authors would put these together. Most of their analysis, was however, performed on Yap and Tap mutants.

Comments for the author

First I apologise for the delay in reviewing this manuscript which has addressed most of the previous comments and presents novel data. I still have a few comments about the current data/presentation as follows.

Introduction
To avoid confusion by readers that are unfamiliar with the field, can the authors make it clear that Yap and Taz are regulated by other factors in addition to the core Hippo pathway. This may explain some of the apparent discrepancy between the two papers that are discussed in the introduction (as well as different stages that were analysed).

Research Data 1.
There was a misunderstanding about my previous comment which I may not have made clear. Figure 1 does not measure proliferation. The data does not show the number of cells – it shows the area that they cover. As the cells are not the same size in the different genotypes, this method can not be used to determine cell number and indirectly, proliferation. The size/shape of the cells also changes during the time course.
In the response to the reviewers comments, the authors state they have also counted individual cells – can this data please be included.
2. The authors state that the Cre has no effect on “proliferation” in the text (Page 5, line 151), but state it has a minor effect in the Figure legend (Fig S1). Please change the text on page 5.
3. Have the authors confirmed the specificity of the Yap and Taz antibodies i.e. by analysis in Yapf/fCre and Tazf/fCre mice? Or can the authors please cite a paper which has confirmed specificity?

© 2020. Published by The Company of Biologists under the terms of the Creative Commons Attribution License (https://creativecommons.org/licenses/by/4.0/).

4. Can the authors confirm that in the proliferation analysis shown in Figure 4 that the number of proliferating cells per total number of cells counted in each zone is being represented. I found it unclear whether it was the number of proliferating cells per unit area that was being analysed.
5. How many sections were analysed per growth plate e.g. in measurements of length, cell density proliferation? (Fig. 4).
6. Are there significant differences between the double mutant and control in E and H, and L in Figure 4 and Fig. 5P? I appreciate the authors could not get additional embryos because of lock-down in response to my previous comment but if the differences between the two sets of data are insignificant (i.e. using unpaired t-test) this should also be stated with numbers of embryos analysed. Would combining the cell density data
(which are effectively the same study done at different times) from Fig. 4E and H yield significance using unpaired t-test?
7. The data in Fig. 4I and J appear inconsistent. Fig. 4I indicates that there must be more cells in the double mutants whilst Fig. 4J indicates no difference. What is the correct data/conclusion?
8. Fig. S5 – the type X collagen domain appears to be expanded in Lats1/2 knockouts which is inconsistent
(opposite) with the constitutively active Yap growth plate. The authors make no comment on this. Lines 381&
382 on p12 are also not representative/as written are slightly misleading.
9. Can the authors comment if Mmp2, Mmp16, and Ctsk have potential Tead binding sites? However, I appreciate that many Yap/Taz binding sites are at enhancer sites making it difficult to identify the gene and Yap/Taz do not always function with Teads but it would be of interest to know if anything can be identified by analysing their promoter sequences.

Minor comments
1. Alter lines 266- 267 on page 8– the cleft palate phenotype is not associated with defects in the cranial base as shown in Fig. 3O.
2. Page 9, lines 291 and 292 are not clear/are incorrect as written as the data shows an increase in the length of the proliferative and hypertrophic region.

Second revision

Author response to reviewers' comments

Reviewer 1 Advance summary and potential significance to field
The manuscript describes the role of Yap and Taz during chondrocyte development using loss of function (double mutant) and gain of function strategies (Yap constitutively active and double Lats1/2 knockout). The authors show that Yap/Taz primarily regulate matrix levels, identifying a few potential regulators/direct targets, and that there is a cleft palate phenotype in the Yap/Taz mutants which is secondary to a defect in Meckel's cartilage.

A change of title may be appropriate. Yap/Taz are not just regulated by the Hippo pathway, although I appreciate that the Lats1/2 mutant shows the same phenotype as constitutively active Yap so I understand why the authors would put these together. Most of their analysis, was however, performed on Yap and Tap mutants.

We appreciate the reviewer's comment, and have now revised the title to mention the "Hippo-Yap/Taz pathway".

Reviewer 1 Comments for the author
First I apologise for the delay in reviewing this manuscript which has addressed most of the previous comments and presents novel data. I still have a few comments about the current data/presentation as follows.

© 2020. Published by The Company of Biologists under the terms of the Creative Commons Attribution License (https://creativecommons.org/licenses/by/4.0/).

Introduction
To avoid confusion by readers that are unfamiliar with the field, can the authors make it clear that Yap and Taz are regulated by other factors in addition to the core Hippo pathway. This may explain some of the apparent discrepancy between the two papers that are discussed in the introduction (as well as different stages that were analysed).

We thank the reviewer for raising this point, and we have added text to the introduction as follows: "In parallel with regulation via LATS1/2 kinases, YAP/TAZ can also be regulated by other inputs, such as direct phosphorylation by Src family kinases (Elbediwy et al., 2018; Elbediwy et al., 2016; Li et al., 2016b; Si et al., 2017)."

Research Data
1.There was a misunderstanding about my previous comment which I may not have made clear. Figure 1 does not measure proliferation. The data does not show the number of cells – it shows the area that they cover. As the cells are not the same size in the different genotypes, this method can not be used to determine cell number and indirectly, proliferation. The size/shape of the cells also changes during the time course. In the response to the reviewers comments, the authors state they have also counted individual cells – can this data please be included.
We now include this data, as requested, in the revised Figure S1.

2.The authors state that the Cre has no effect on "proliferation" in the text (Page 5, line 151), but state it has a minor effect in the Figure legend (Fig S1). Please change the text on page 5.
Fixed.

3.Have the authors confirmed the specificity of the Yap and Taz antibodies i.e. by analysis in Yapf/fCre and Tazf/fCre mice? Or can the authors please cite a paper which has confirmed specificity?
We now include this citation (Elbediwy et al 2016) in the methods section. Note that we also show the specificity of these antibodies in our knockout growth plates in Figure S3.

4.Can the authors confirm that in the proliferation analysis shown in Figure 4 that the number of proliferating cells per total number of cells counted in each zone is being represented. I found it unclear whether it was the number of proliferating cells per unit area that was being analysed.
To clarify this point, we have amended the text describing this analysis in the results section (p9) to point out that it is the percentage of proliferating cells (of the total number of cells marked by Eosin or DAPI in each area).

5.How many sections were analysed per growth plate e.g. in measurements of length, cell density, proliferation? (Fig. 4).
This information is provided in the relevant figure legend. It is also evident in the data points on the graphs.

6.Are there significant differences between the double mutant and control in E and H, and L in Figure 4 and Fig. 5P? I appreciate the authors could not get additional embryos because of lock-down in response to my previous comment but if the differences between the two sets of data are insignificant (i.e. using unpaired t-test) this should also be stated with numbers of embryos analysed. Would combining the cell density data (which are effectively the same study done at different times) from Fig. 4E and H yield significance using unpaired t-test?
We have used a 2-way ANOVA to measure statistical significance in these experiments.

7.The data in Fig. 4I and J appear inconsistent. Fig. 4I indicates that there must be more cells in the double mutants whilst Fig. 4J indicates no difference. What is the correct data/conclusion?
The data in 4I show very subtle differences, which vary along the length of the growth plate. The data in 4J do show more cells in the double knockout (the mean is higher), but the difference is so subtle as to not reach statistical significance along the entire growth plate. This is all consistent with our conclusions.

8.Fig. S5 – the type X collagen domain appears to be expanded in Lats1/2 knockouts which is inconsistent (opposite) with the constitutively active Yap growth plate. The authors make no

© 2020. Published by The Company of Biologists under the terms of the Creative Commons Attribution License (https://creativecommons.org/licenses/by/4.0/).

comment on this. Lines 381& 382 on p12 are also not representative/as written are slightly misleading.
We don't quite agree that the data in Fig S5 show an expanded area of COLX staining in Lats1/2 KO compared to the controls, instead it appears either smaller or abnormally shaped. We have amended the relevant text to comment on this point. Importantly, the reviewer seems to agree with our general point the YAP activation (in Lats1/2 KO or otherwise) does not simply function by directly repressing type X collagen expression, as claimed by others. We add a sentence to the text to state that "these effects may be a secondary consequence of the highly abnormal morphology of these growth plates."

9.Can the authors comment if Mmp2, Mmp16, and Ctsk have potential Tead binding sites? However, I appreciate that many Yap/Taz binding sites are at enhancer sites making it difficult to identify the gene and Yap/Taz do not always function with Teads but it would be of interest to know if anything can be identified by analysing their promoter sequences.
It is very difficult to rule-in or rule-out TEAD binding sites as they can be located within enhancers many kilobases away from the promoter start site in either direction. A full analysis of the enhancers governing these genes is unfortunately outside the scope of this manuscript.

Minor comments
1.Alter lines 266- 267 on page 8– the cleft palate phenotype is not associated with defects in the cranial base as shown in Fig. 30.
We do see a wider cranial base in all three classes of mutants compared to controls (Fig 3K-N), so we would prefer to keep this sentence as it currently is.

2.Page 9, lines 291 and 292 are not clear/are incorrect as written as the data shows an increase in the length of the proliferative and hypertrophic region.
Fixed.

Reviewer 3 Advance summary and potential significance to field
The authors have reasonably addressed some of the comments. In addition, the suggestion to use Col2-CreER mice requires a considerable amount of work. However, what I fail to understand is the authors' rejection of the suggestion to observe their mice at earlier stages of development. In contrast to the authors' claims in the rebuttal letter, without such examination it is impossible to discern between direct effects of early deletion of YAP/TAZ and secondary consequences. Therefore, without these data I cannot fully support publication.
Reviewer 3 Comments for the author
The authors have reasonably addressed some of the comments. Also, the suggestion to use Col2-CreER mice requires a considerable amount of work. However, what I fail to understand is the authors' rejection of the suggestion to observe their mice at earlier stages of development. In contrast to the authors' claims in the rebuttal letter, without such examination, it is impossible to discern between direct effects of early deletion of YAP/TAZ and secondary consequences. Therefore, without these data, I cannot fully support publication.

To address this point, we have amended the text to state that "we note that phenotyping at E17.5 does not discern the direct early effects of YAP/TAZ deletion and that, to address this issue, earlier stages of development would need to be examined." (Results line 213-214).

Third decision letter

MS ID#: DEVELOP/2019/187187

MS TITLE: Control of skeletal morphogenesis by the Hippo-YAP/TAZ pathway

AUTHORS: Hannah Vanyai, Fabrice Prin, Oriane Guillermin, Bishara Marzook, Stefan Boeing, Alexander Howson, Rebecca Saunders, Thomas Snoeks, Michael Howell, Timothy J Mohun, and Barry Thompson

© 2020. Published by The Company of Biologists under the terms of the Creative Commons Attribution License (https://creativecommons.org/licenses/by/4.0/).

ARTICLE TYPE: Research Article

I am happy to tell you that your manuscript has been accepted for publication in Development, pending our standard ethics checks.

© 2020. Published by The Company of Biologists under the terms of the Creative Commons Attribution License (https://creativecommons.org/licenses/by/4.0/).
